# Computational carrier dynamics across heterojunction interface between hole injection and transport layers in quantum-dot light-emitting diodes

**DOI:** 10.1038/s41598-026-55391-2

**Published:** 2026-06-16

**Authors:** Sung-Min Jung, Yoonwoo Kim, Jeong-Wan Jo, Dean M. Thelen, Michal Mlejnek, Caicai Zhang, Jong Min Kim

**Affiliations:** 1 Electrical Engineering Division, Department of Engineering, University of Cambridge, 9 JJ Thomson Ave, Cambridge, CB3 0FA UK; 2 Corning Research and Development Corporation, Corning, NY 14831 USA; 3 Corning Incorporated, Corning, NY 14831 USA; 4 Present Address: Department of Electronics Engineering, Incheon National University, Incheon, 22012 Republic of Korea; 5 Present Address: AllFocal Optics, St John’s Innovation Centre, Cambridge, CB4 0WS UK

**Keywords:** Drift–diffusion modelling, Heterojunction interfaces, Numerical stability, Quantum-dot light-emitting diodes, Charge transport simulation, Engineering, Materials science, Nanoscience and technology, Optics and photonics, Physics

## Abstract

Physics-based charge transport modelling is widely used to analyse multilayer optoelectronic devices. However, conventional drift–diffusion discretisation schemes can exhibit numerical instability at heterojunction interfaces with abrupt discontinuities in energy levels and doping density. In quantum-dot light-emitting diodes (QD-LEDs), the heterojunction between the hole injection layer (HIL) and the hole transport layer (HTL) represents such a critical interface. In this study, a field-dependent current density scheme is proposed to stabilise the discretisation of drift–diffusion currents across heterojunction interfaces. By incorporating the local electric-field direction when evaluating carrier densities at discretised boundaries, the scheme suppresses numerical artefacts associated with mean-value interpolation of the carrier density. The stability and convergence of our model are examined using a one-dimensional finite-difference framework and subsequently implemented in a charge transport model for QD-LEDs. Using this model, the effects of energy-level alignment and acceptor doping density at the HIL/HTL interface on charge transport and electro-optical characteristics are analysed. The simulations reproduce typical voltage-dependent experimental trends in current density, luminance, and external quantum efficiency, providing insight into the role of the HIL/HTL heterojunction in carrier injection. Owing to its numerical formulation, the proposed approach is applicable to a broad range of multilayer semiconductor devices involving heterojunction interfaces.

**Supplementary Information:**

The online version contains supplementary material available at https://doi.org/10.1038/s41598-026-55391-2.

## Introduction

Physics-based charge transport modelling based on the drift–diffusion formalism remains a cornerstone for the analysis and design of modern semiconductor devices^1–4^. Owing to its physical fidelity, the drift–diffusion framework has been widely employed to investigate carrier transport in multilayer electronic and optoelectronic systems, including organic light-emitting diodes (OLEDs), quantum-dot light-emitting diodes (QD-LEDs), and thin-film transistors^5–16^. However, when drift–diffusion-based simulations are applied to devices incorporating heterojunction interfaces with abrupt discontinuities in material parameters, achieving numerical stability and physically meaningful solutions becomes inherently challenging^17^.

In QD-LED architectures, the heterojunction between the hole injection layer (HIL) and the hole transport layer (HTL) plays a critical role in governing carrier injection, accumulation, and recombination^18–20^. The HIL/HTL heterojunction interface can be assumed by an abrupt change in energy band alignment and doping concentration, and this interface is typically characterised by sharp offsets in energy levels, spatially varying doping density, and strong internal electric fields. Conventional drift–diffusion discretisation schemes in numerical modelling commonly evaluate carrier densities at the interfaces using arithmetic or logarithmic mean-value approximations^12,21^. While such approaches are generally adequate in homojunction regions with slowly varying potentials, applying conventional discretisation schemes at heterojunction interfaces with strong field gradients often resulted in oscillatory or poorly convergent carrier profiles in our numerical experiments^22^.

A variety of numerical strategies have been explored to improve stability at heterojunction interfaces, including refined mesh discretisation, the Scharfetter–Gummel (SG) scheme based on interpolated Bernoulli-function exponential fitting, and interface-specific boundary treatments^23–26^. The SG scheme, in particular, provides a widely adopted and numerically stable discretisation of drift–diffusion transport by assuming a linear variation of the electrostatic potential within each discretisation cell, and is well suited for systems with continuous (albeit potentially steep) carrier density distributions. Such SG-type discretisation schemes effectively evaluate carrier transport based on quantities defined across adjacent discretisation nodes through exponential fitting. However, such approaches become less appropriate at abrupt heterojunction interfaces, where discontinuities in both band alignment and carrier density arise. Despite their effectiveness in specific scenarios, these approaches may require problem-dependent tuning parameters or employ numerical smoothing at heterojunction interfaces, which has been reported to compromise the physical fidelity of the simulated carrier transport^21,27,28^. More broadly, Commercial technology computer-aided design (TCAD) tools model heterojunctions within the drift–diffusion framework, sometimes supplemented by alternative transport models (e.g., hopping-based charge transfer) or internal numerical treatments. However, numerical instabilities associated with discontinuities in band alignment and carrier density can still arise in practical device simulations, depending on the discretisation and modelling assumptions. Consequently, a discretisation strategy that remains numerically stable while preserving physical transparency is still desirable, particularly for multilayer device structures involving abrupt heterojunctions.

In this work, a field-dependent carrier density selection scheme is introduced to stabilise the discretisation of drift–diffusion currents across the HIL/HTL heterojunction in QD-LED devices. The proposed approach explicitly accounts for the local electric-field direction when evaluating carrier densities at discretised cell boundaries, enabling a physically consistent upwind selection of carrier populations that is applied locally at the heterojunction interface rather than as a global numerical stabilisation scheme. This localised treatment enables stable and physically consistent current evaluation in the presence of discontinuities in band alignment and strongly asymmetric carrier distributions. The scheme is incorporated into a physics-based charge transport model for QD-LEDs. The numerical behaviour of the scheme is first examined within a one-dimensional finite-difference framework to assess its stability and convergence characteristics. Subsequently, heterojunction-limited hole injection and its dependence on energy-level alignment and HIL doping density are quantitatively examined.

Overall, the results demonstrate that incorporating local electric-field information into the discretisation of carrier densities provides a stable and physically consistent description of charge transport across heterojunction interfaces. While the HIL/HTL interface in QD-LEDs is used here as a representative case, the underlying formulation is not restricted to a specific material system or device architecture and may be readily extended to a broad range of multilayer semiconductor devices in which abrupt heterojunctions play a central role.

## Results

### Device architecture and layer configuration of QD-LEDs

**Fig. 1:**
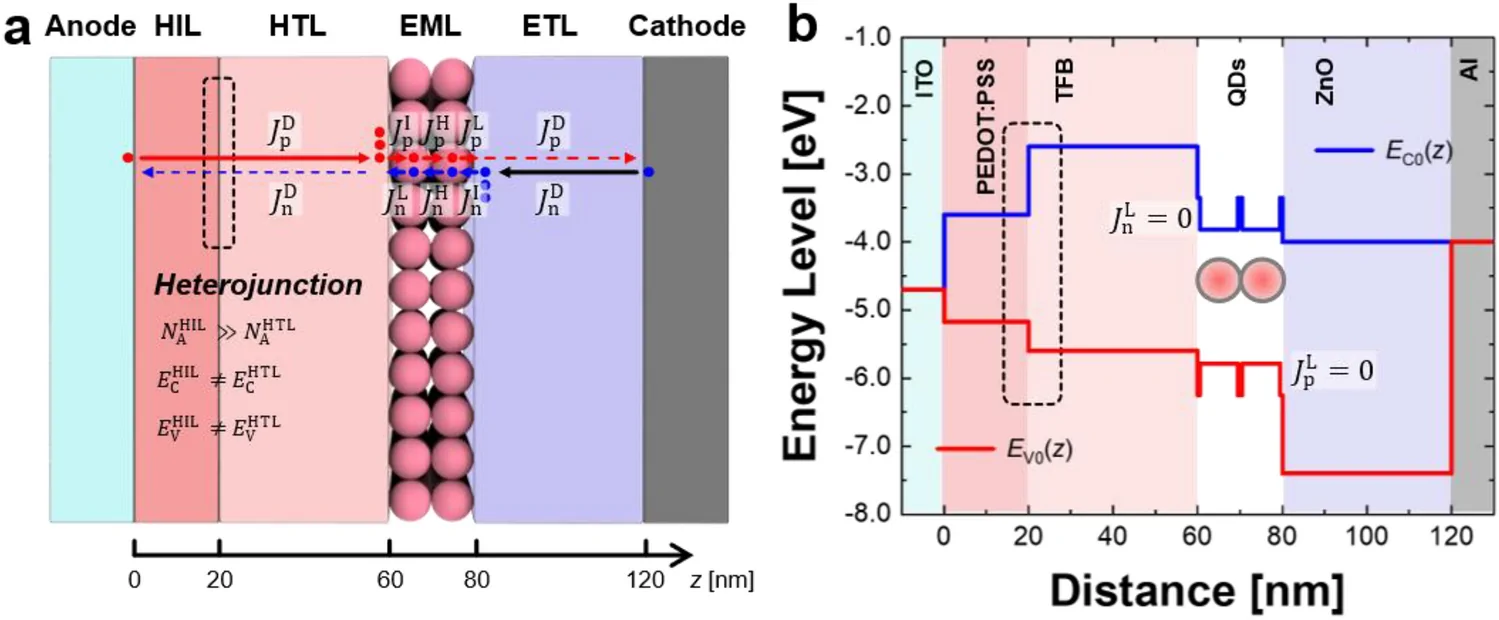
Schematic representation of the QD-LED device configuration used for charge transport modelling. (**a**) Multilayer device structure consisting of a hole injection layer (HIL), hole transport layer (HTL), quantum-dot-based emissive layer (EML), and electron transport layer (ETL), positioned between anode and cathode electrodes. Representative current pathways, such as drift–diffusion *J*^D^, injection *J*^I^, and hopping *J*^H^ current densities, are indicated for both holes and electrons. (**b**) Flat-band energy level diagram of the QD-LED device layers, showing the band edges *E*_V0_(*z*) and *E*_C0_(*z*). A heterojunction is located at the HIL/HTL interface (*z* = 20 nm), where the valence band energies of the HIL and HTL are set to − 5.17 eV and − 5.60 eV, respectively, and the conduction band energies are set to − 3.60 eV and − 2.60 eV, respectively. The acceptor doping concentrations are 2.81 × 10^19^ cm^−3^ for the HIL and 1.00 × 10^17^ cm^−3^ for the HTL.

The device architecture used for charge transport modelling in the QD-LED is illustrated in Fig. 1. The device structure comprises multiple nanolayers, including a HIL, an HTL, an EML and an ETL, sequentially stacked between anode and cathode electrodes (Fig. 1a). The EML is modelled as an *M* number of QD layers (Here,* M* = 2 for a double layer of QDs in the figure). Possible current pathways across the device are schematically shown in Fig. 1a. Upon applying an external voltage across the anode and cathode, holes and electrons are transported by drift–diffusion through the HIL/HTL and ETL, respectively, toward both boundaries of EML. These carriers are then injected into the QD layer via an electric-field-dependent carrier capture process and subsequently propagate through adjacent QDs via a carrier hopping process. Accordingly, three types of current densities are modelled for holes and electrons: (i) drift–diffusion current densities *J*_p_^D^ and *J*_n_^D^ in the HIL/HTL and ETL transport layers (where subscripts p and n refer to holes and electrons); (ii) injection current densities *J*_p_^I^ and *J*_n_^I^ at the HTL/EML and EML/ETL interfaces; and (iii) carrier hopping current densities *J*_p_^H^ and *J*_n_^H^ among neighbouring QDs. Carrier recombination within the EML, involving accumulated electron–hole pairs, occurs through radiative Langevin recombination, and non-radiative SRH and Auger recombination processes.

The flat-band energy level diagram of the conduction band, *E*_C0_(*z*), and valence band, *E*_V0_(*z*), for the multilayer structure along the device distance *z* from the anode/HIL interface is depicted in Fig. 1b. The materials used in this study for the anode, HIL, HTL, EML, ETL, and cathode are indium tin oxide (ITO), poly(3,4-ethylenedioxythiophene) polystyrene sulfonate (PEDOT:PSS), poly [(9,9-dioctylfluorenyl-2,7-diyl)-co-(4,4′-(N-(4-s-butylphenyl)diphenylamine)] (TFB), QDs, zinc oxide (ZnO), and aluminium (Al), respectively. The thicknesses of PEDOT:PSS, TFB, and ZnO are assumed to be 20 nm, 40 nm, and 40 nm, respectively, while the EML consists of two 10 nm QD layers, totalling 20 nm in thickness^16^.

In the figure, three heterojunctions are identified in the device: (i) HTL/EML interface at *z* = 60 nm, (ii) EML/ETL interface at *z* = 80 nm, and (iii) HIL/HTL interface at *z* = 20 nm. At the HTL/EML and EML/ETL interfaces, carrier injection into the QD layer is governed by electric-field-dependent carrier capture across valence and conduction band offsets. For simplicity, the valence and conduction band offsets at both interfaces are assumed to be identical. Additionally, the electron leakage current density *J*_n_^L^ at the HTL/EML interface and the hole leakage current density *J*_p_^L^ at the EML/ETL interface were set to 0 A cm^−2^, reflecting the hole and electron blocking functions of the HTL and ETL, respectively.

At the HIL/HTL interface (*z* = 20 nm), a distinct heterojunction is formed due to mismatches in the flat-band energy levels and carrier doping densities. Specifically, the valence band energies of PEDOT:PSS (HIL), *E*_V_^HIL^, and TFB (HTL), *E*_V_^HTL^, are set to −5.17 eV and −5.60 eV, respectively, resulting in a valence band offset of approximately 0.43 eV for the hole. The conduction band energies are −3.60 eV (HIL), *E*_C_^HIL^, and −2.60 eV (HTL), *E*_C_^HTL^, yielding a 1.0 eV offset for the electron. Moreover, the acceptor doping density *N*_A_^HIL^ in the HIL is set to 2.81 × 10^19^ cm^−3^, which is significantly higher than that of the HTL, *N*_A_^HTL^ = 1.0 × 10^17^ cm^−3^. Material and device parameters for the transport layers and QDs are summarised in Tables S1 and S2. In this study, hole transport in PEDOT:PSS (HIL) and TFB (HTL) is treated within the drift–diffusion formalism, which has been widely applied to doped organic semiconductors owing to their effectively band-like transport behaviour at relevant doping levels^29,30^. Since carrier transport in the HIL and HTL regions is governed by the drift–diffusion model, the discontinuities in energy levels and the doping densities at the heterojunction must be carefully treated to ensure accurate and stable numerical simulation.

### Discretisation of the continuity equation

**Fig. 2:**
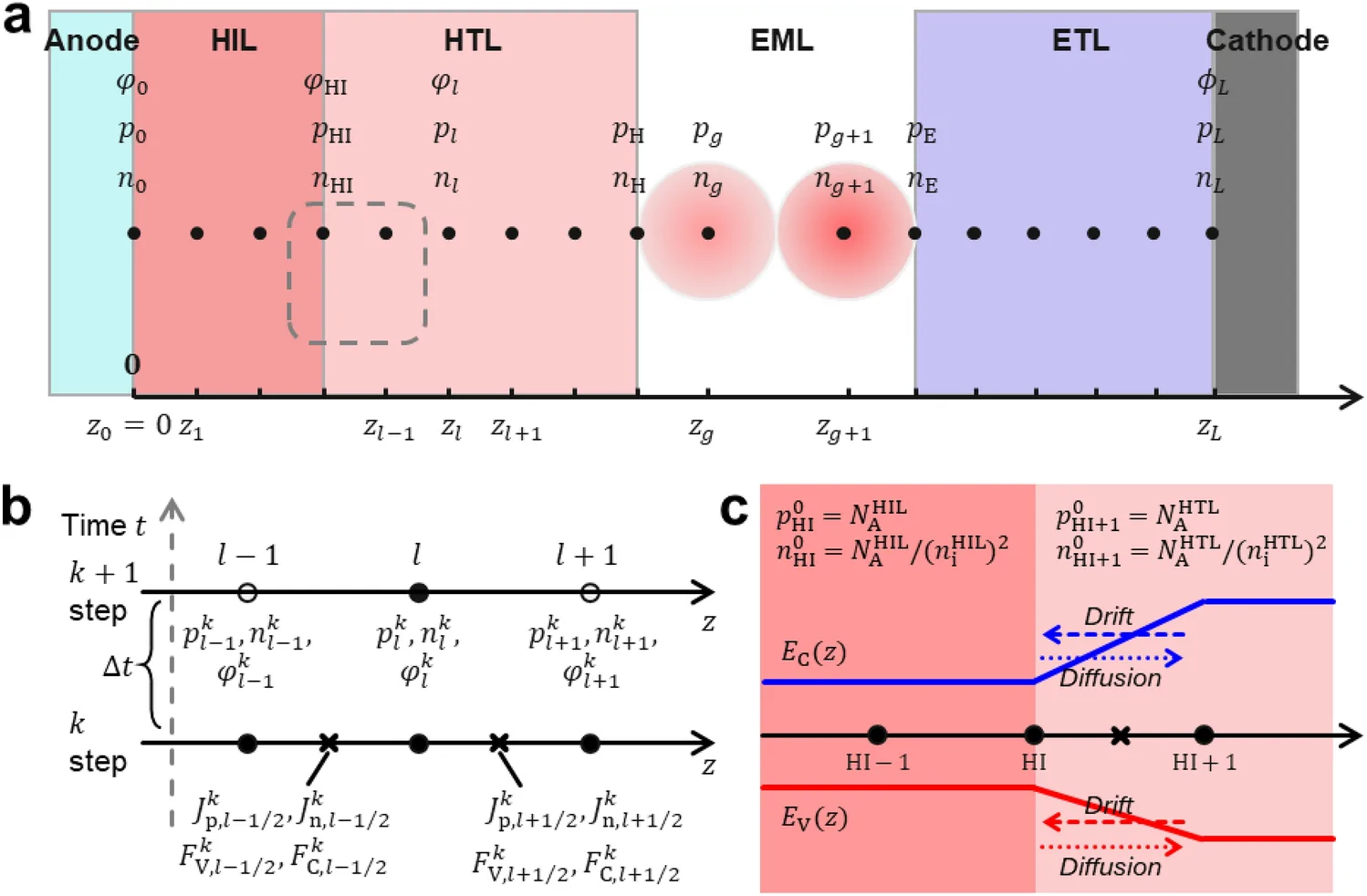
(**a**) Numerical grid on the computational domain. (**b**) Finite difference schemes in time and spatial domain with nodal definition of the numerical parameters. (c) Discontinuity of doping density and the valence band energy level at the heterojunction interface between HIL and HTL.

Charge carrier transport within the QD-LED device is governed by the continuity equation, which is a set of nonlinear, time-dependent, second-order partial differential equations. The detailed charge transport model for the QD-LED device is described in the Methods section. In this study, the finite difference method (FDM) was employed to numerically solve the continuity equation on a discretised computational domain. Figure 2 illustrates the computational domain used for the QD-LED simulation. As shown in Fig. 2a, the spatial domain is inhomogeneously discretised with nodal spacing *z*_*l*_ indexed by integer *l* (0 ≤ *l* ≤ *L*). The boundary conditions for carrier densities and electrostatic potential are applied at nodes* l* = 0 and *l* = *L*. The EML is discretised at the centres of the QD layers to reflect the localised carrier states^12^, where *g* denotes the first grid index in the EML region following the HIL/HTL grids.

Figure 2b illustrates the explicit time discretisation, where *t* denotes the time variable and is updated by *t* = Δ*t* × *k* at a given *k*-th time step, using a given time interval Δ*t*. The hole and electron carrier densities at the (*k* + 1)-th time step and the *l*-th spatial grid point, *p*_*l*_^*k*+1^ and *n*_*l*_^*k*+1^, are calculated by using the discretised continuity equations in Eq. (1) from the data given at *k*-th time step.

1 $$\begin{gathered} p_{l}^{k + 1} = p_{l}^{k} + \Delta t\left[ { - \frac{{J_{\mathrm{p},l + 1/2}^{k} - J_{\mathrm{p},l - 1/2}^{k} }}{{q\left( {z_{l + 1} - z_{l - 1} } \right)/2}} - U_{l}^{k} } \right], \hfill \\ n_{l}^{k + 1} = n_{l}^{k} + \Delta t\left[ {\frac{{J_{\mathrm{n},l + 1/2}^{k} - J_{\mathrm{n},l - 1/2}^{k} }}{{q\left( {z_{l + 1} - z_{l - 1} } \right)/2}} - U_{l}^{k} } \right]. \hfill \\ \end{gathered}$$

Here, *p*_*l*_^*k*^ and *n*_*l*_^*k*^ are the hole and electron carrier densities at the *l*-th grid point and *k*-th time step, and the *J*_p,*l*±1/2_^* k*^ denote the hole current densities evaluated at the midpoints between two adjacent nodes. *q* is the elementary charge, and *z*_*l*+1_ and *z*_*l*−1_ are the positions of (*l* + 1)-th and (*l*−1)-th nodal points. *U*_*l*_^*k*^ denotes the total recombination rate at the *l*-th grid point defined in the Methods section.

Figure 2c depicts the local structure around the HIL/HTL heterojunction interface, where abrupt changes in energy levels and doping density occur. In this model, node *l* = HI is assigned to the HIL region and node *l* = HI + 1 to the HTL region. The drift–diffusion current densities between these two nodes for hole and electron, *J*_p,HI+1/2_^D,*k*^ and *J*_n,HI+1/2_^D,*k*^ at k-th time step are discretised using Eq. (2), with the grid spacing Δz_HTL_ = *z*_HI+1_ – *z*_HI_ defined on the HTL side.

2 $$\begin{gathered} J_{\text{p,HI + 1/2}}^{\mathrm{D}, k} = q\mu_{\mathrm{p}} p_{\text{HI + 1/2}}^{k} F_{\text{V,HI + 1/2}} - \mu_{\mathrm{p}} k_{\mathrm{B}} T\frac{{p_{\text{HI + 1}}^{k} - p_{\mathrm{HI}}^{k} }}{{\Delta z_{\mathrm{HTL}} }} \hfill \\ J_{\text{n,HI + 1/2}}^{\mathrm{D}, k} = q\mu_{\mathrm{n}} n_{\text{HI + 1/2}}^{k} F_{\text{C,HI + 1/2}} + \mu_{\mathrm{n}} k_{\mathrm{B}} T\frac{{n_{\text{HI + 1}}^{k} - n_{\mathrm{HI}}^{k} }}{{\Delta z_{\mathrm{HTL}} }} \hfill \\ \end{gathered}$$

Here, *µ*_p_ and *µ*_n_ are the hole and electron mobilities in the HTL region, and *p*_HI+1/2_^* k*^ and *n*_HI+1/2_^* k*^ denote the hole and electron densities, respectively, at the midpoint between the HI and HI + 1 nodes. *k*_B_ and *T* are the Boltzmann constant and the absolute temperature of the device. These two terms in Eq. (2) correspond to the drift and diffusion components of the current density. The electric-field-related terms on the valence and conduction band edges, *F*_V,HI+1/2_ and *F*_C,HI+1/2_, are defined as *F*_V,HI+1/2_ = (*E*_V0_^HIL^ ‒ *E*_V0_^HTL^)/(*q*Δ*z*_HTL_) ‒ (*φ*_HI+1_ ‒ *φ*_HI_)/Δ*z*_HTL_ and *F*_C,HI+1/2_ = (*E*_C0_^HIL^ – *E*_C0_^HTL^)/(*q*Δ*z*_HTL_) ‒ (*φ*_HI+1_ ‒ *φ*_HI_)/Δ*z*_HTL_, respectively, for the flat-band valence and conduction band energy levels, *E*_V0_^HIL^, *E*_C0_^HIL^, *E*_V0_^HTL^ and *E*_C0_^HTL^, of the HIL and HTL and the discretised potential values, *φ*_HI_ and *φ*_HI+1_ for HI and HI + 1 nodes, solved numerically by Poisson’s equation (Methods section).

Due to the band alignment and doping profiles at the HIL/HTL interface, holes and electrons experience drift from the HTL toward the HIL (reverse direction), while diffusion occurs from the HIL toward the HTL (forward direction). These opposing currents balance out over time, leading to a steady-state solution. Notably, a higher acceptor doping concentration in the HIL is essential for achieving this balance with the drift of holes in the reverse direction, as hole transport dominates in these *p*-type materials.

One option for evaluating the midpoint carrier densities *p*_HI+1/2_ and *n*_HI+1/2_ in the first terms of Eq. (2) is to take the arithmetic mean: *p*_HI+1/2_ = (*p*_HI_ + *p*_HI+1_)/2 and *n*_HI+1/2_ = (*n*_HI_ + *n*_HI+1_)/2. This is referred to as the mean value carrier density scheme. Alternatively, the carrier density at the midpoint can be referred to as the electric-field-dependent carrier density selection scheme as described in Eqs. (3) and (4).

3 $$p_{\mathrm{HI+1/2}} = \left\{ {\begin{array}{*{20}c} { p_{\mathrm{HI+1}} , F_{\mathrm{V,HI+1/2}} < 0} \\ {p_{\mathrm{HI}} , F_{\mathrm{V,HI+1/2}} \ge 0} \\ \end{array} } \right. ,$$

4 $$n_{{{\mathrm{HI}} + 1/2}} = \left\{ {\begin{array}{*{20}c} { n_{{{\mathrm{HI}}}} , F_{{{\mathrm{C}},{\mathrm{HI}} + 1/2}} < 0} \\ {n_{{{\mathrm{HI}} + 1}} , F_{{{\mathrm{C}},{\mathrm{HI}} + 1/2}} \ge 0} \\ \end{array} } \right.$$

**Fig. 3:**
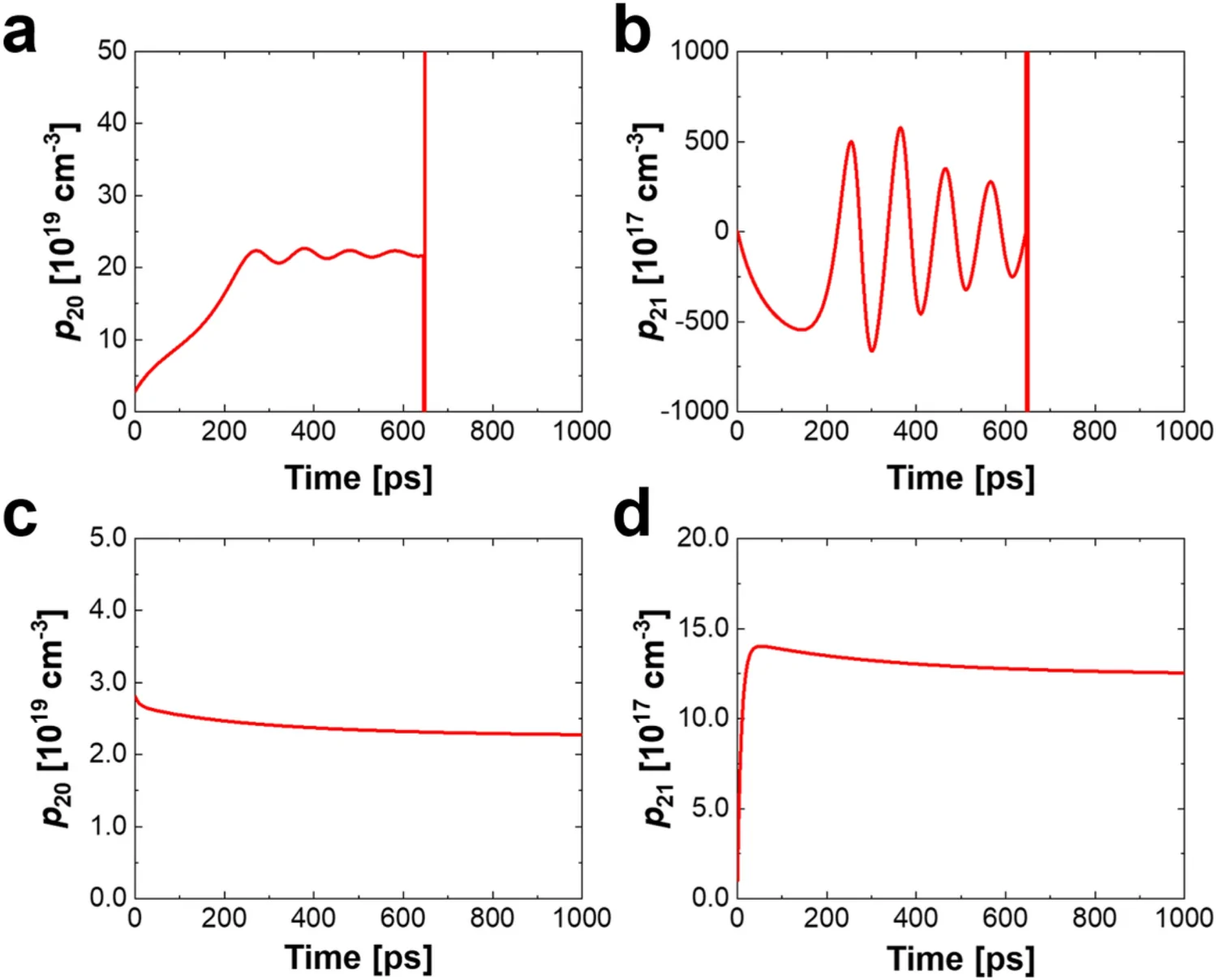
Time evolution of hole densities at the HIL/HTL interface (HI = 20) under two discretisation schemes. Instability observed using the mean value carrier density scheme: (**a**) *p*_20_ increases with oscillations, and (**b**) *p*_21_ diverges after undershooting. Stable convergence was achieved using the electric-field-dependent carrier density selection scheme: (**c**) *p*_20_ and (d) *p*_21_.

Figure 3 presents the time-dependent evolution of hole densities at the HIL/HTL interface to investigate the numerical stability of both schemes during the time integration of Eq. (1) with the fixed time step Δ*t* = 1 ps. The interfacial node index HI = 20 corresponds to the HIL/HTL junction, assuming a uniform grid spacing of 1 nm within the 20 nm-thick HTL. The initial hole densities at *p*_20_ and *p*_21_ are 2.81 × 10^19^ cm^−3^ and 1.00 × 10^17^ cm^−3^, respectively. The corresponding initial electron densities *n*_20_ and *n*_21_ are computed to 0.94 × 10^–7^ cm^−3^ and 2.53 × 10^–29^ cm^−3^ using the mass action law, *pn* = *n*_i_^2^, with intrinsic carrier densities *n*_i_ = 1.63 × 10^6^ cm^−3^ and 1.59 × 10^–6^ cm^−3^ for the respective HIL and HTL (Table S1).

When the mean value carrier density scheme is applied, the hole density *p*_20_ exhibits numerical oscillation after an initial linear increase over time (Fig. 3a), while *p*_21_ initially drops to a negative value and then also shows strong oscillatory behaviour (Fig. 3b). Subsequently, both values abruptly diverge after *t* = 650 ps, indicating numerical instability. Since *p*_20_ ≫ *p*_21_, the midpoint hole density *p*_20.5_ is numerically dominated by *p*_20_, leading to an overestimated outward drift current when updating *p*_21_, particularly under a negative electric field. This causes an unphysical undershoot of *p*_21_ below zero (see also Figs. S1a and S1b for the electron case). In contrast, the field-dependent carrier density selection scheme yields stable convergence for both *p*_20_ and *p*_21_ (Figs. 3c and 3 d), as the midpoint value *p*_20.5_ is set to *p*_21_ under negative field conditions. This prevents overestimation of the drift current and avoids negative carrier density (see also Figs. S1c and S1d for the electron case). These results demonstrate that the field-dependent carrier selection scheme provides a more numerically stable approach for treating carrier density across the heterojunction interface, taking into account the energy band alignment and doping profile of the HIL and HTL in QD-LED device structures. Moreover, the proposed field-dependent carrier density selection scheme preserves charge conservation, as the continuity equation is discretised in a flux-conservative form. The interfacial current density is uniquely defined at each cell boundary and is shared by the two adjacent nodes with opposite signs, ensuring that the current leaving one node is exactly equal to the current entering the neighbouring node. Therefore, the present scheme modifies only the evaluation of the interfacial drift current without altering the underlying charge balance across the heterojunction interface.

### Numerical charge transport simulation of HOD

**Fig. 4:**
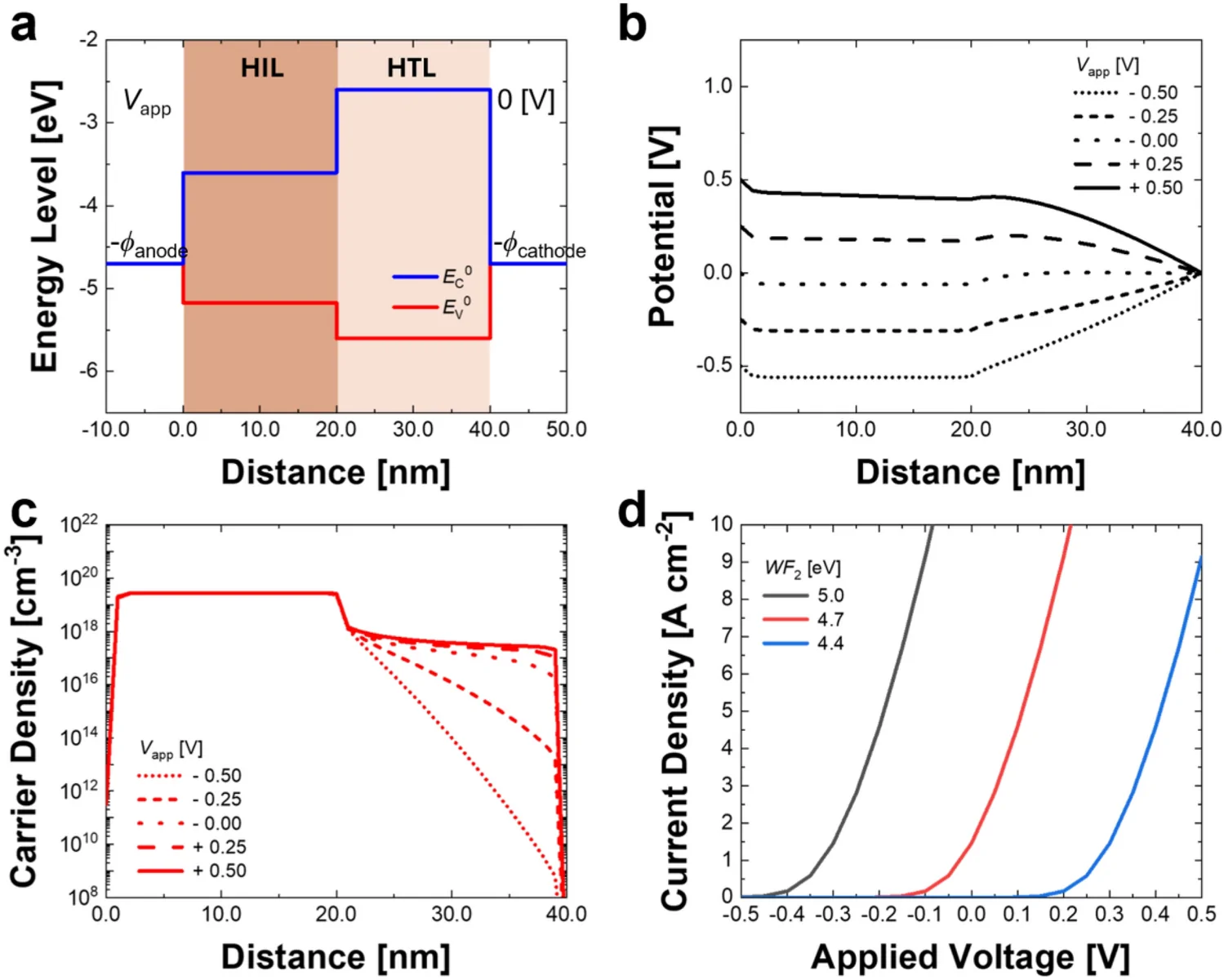
Simulated charge transport characteristics of the hole-only device (HOD) comprising anode/PEDOT:PSS (20 nm)/TFB (20 nm)/cathode. (**a**) Flat-band energy diagram highlighting the abrupt discontinuity at the HIL/HTL interface. (**b**) Simulated potential profiles under applied biases of –0.5 V, –0.25 V, 0 V, + 0.25 V, and + 0.5 V. (**c**) Corresponding hole density distributions across the HTL under forward and reverse biases. (**d**) Current–voltage characteristics as a function of the cathode work function, showing diode-like rectification behaviour and barrier-limited injection.

To further validate the field-dependent carrier selection model for drift current density at the HIL/HTL heterojunction in isolation from the full QD-LED stack, we additionally established a computational charge transport model for an HOD. Using the simplified bilayer structure of anode/PEDOT:PSS(HIL)/TFB(HTL)/cathode, we performed drift–diffusion simulations of the voltage-dependent potential distribution, carrier density profiles, and current–voltage characteristics. Figure 4 presents the simulated electrical properties of the HOD device with varying applied voltage and cathode work function. The thicknesses of the PEDOT:PSS and TFB were both fixed at 20 nm, and the acceptor doping densities *N*_A_ of the PEDOT:PSS and TFB were set to be 2.81 × 10^1^⁹ cm⁻^3^ and 1.0 × 10^1^⁷ cm⁻^3^, respectively, identical to those in full QD-LED device modelling. The work functions of the anode and cathodes are defined by *ϕ*_anode_ and *ϕ*_cathode_, respectively.

In Fig. 4a, the flat-band energy diagram shows the abrupt discontinuity in energy levels and carrier densities at the HIL/HTL interface. Figure 4b presents the simulated potential profiles under applied biases of –0.5 V, –0.25 V, 0 V, + 0.25 V, and + 0.5 V. Owing to its high hole doping density, the HIL acts nearly as a metallic conductor with negligible potential drop, whereas most of the voltage drop occurs across the HTL region. Under forward bias, the potential gradient in the HTL becomes negative, enabling hole transport from the HIL toward the cathode; under reverse bias, the potential gradient becomes positive, suppressing hole transport across the HTL. Correspondingly, Fig. 4c shows that the hole density in the HTL is strongly reduced under reverse bias (–0.5 V), whereas under forward bias (+ 0.5 V), a uniform and sufficient hole density is established across the HTL, enabling efficient hole conduction.

Finally, Fig. 4d presents the simulated current–voltage characteristics as a function of the cathode work function (*ϕ*_cathode_). The results clearly exhibit diode-like rectification: the current remains negligible below a threshold voltage and increases sharply once the applied voltage exceeds the injection barrier. Increasing the cathode work function enlarges the hole injection barrier, shifting to a higher threshold voltage, consistent with barrier-limited injection physics. These results demonstrate that the proposed field-dependent drift current model accurately describes charge transport in a simplified HIL/HTL bilayer device with discontinuities in band alignment and carrier density. Importantly, this provides direct numerical evidence that the scheme remains stable and physically meaningful even without including the QD and ETL layers.

### Numerical charge transport simulation of full-stack QD-LED devices

**Fig. 5:**
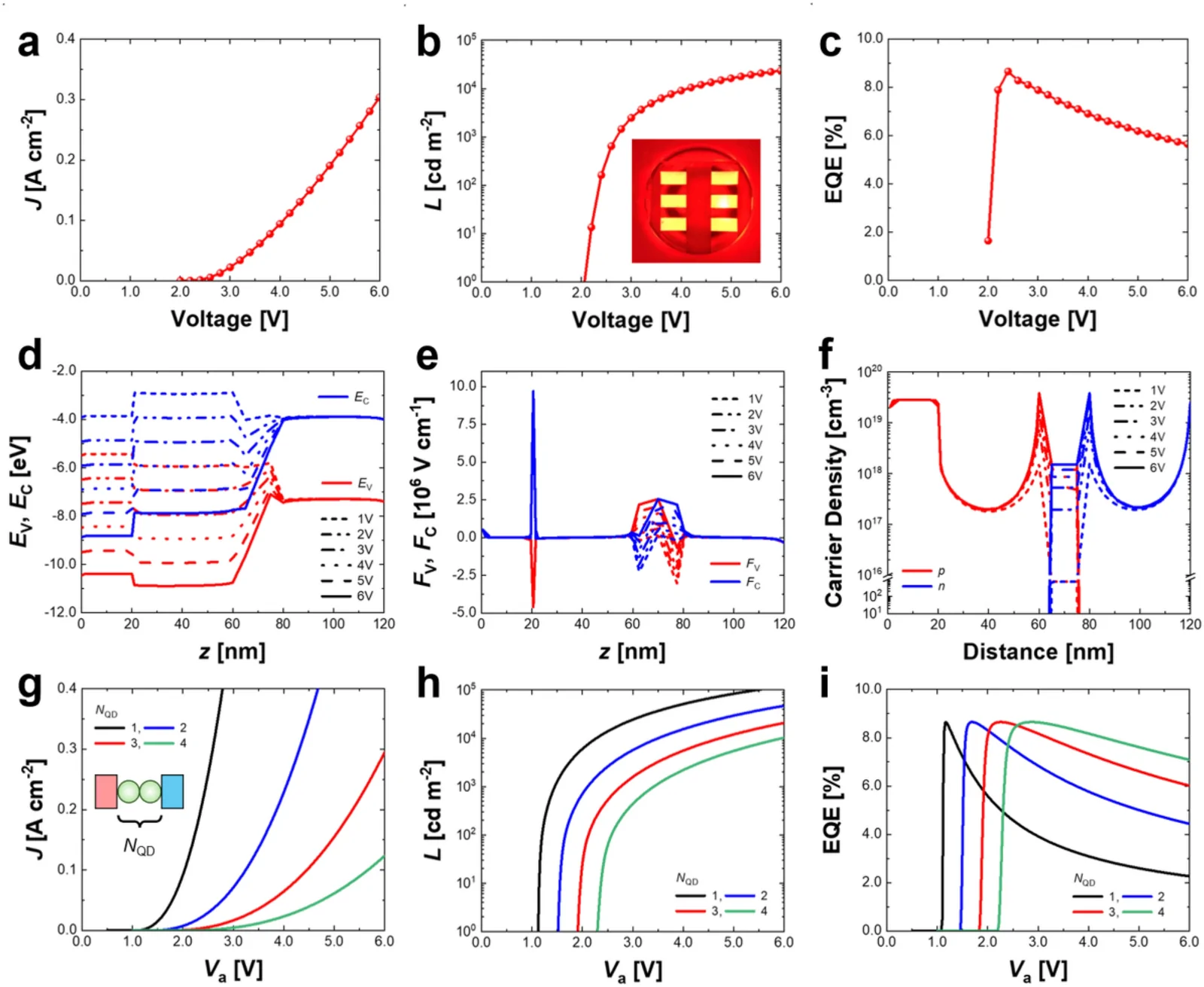
Comparison between the experimental and simulated electro-optical characteristics of QD-LED devices. Experimental voltage-dependent characteristics of (**a**) current density, (**b**) luminance with an inset for device snapshot, and (**c**) EQE for the CdSe/ZnS red QD-LED device. Simulated charge transport characteristics in a full QD-LED device using a field-dependent selection scheme for drift current modelling at the HIL/HTL heterojunction under applied voltages from 1 to 6 V (in 1 V increments) for different numbers of QD layers (*N*_QD_) (d-i). (**d**) Valence (red) and conduction (blue) band energy levels coupled with the electrostatic potential distribution obtained from Poisson’s equation when *N*_QD_ is 2. (**e**) Electric field distributions in the valence (red) and conduction (blue) bands across the device. (**f**) Spatial distributions of hole (red) and electron (blue) densities. Simulated voltage-dependent device characteristics of (g) current density, (h) luminance, and (i) EQE for *N*_QD_ = 1, 2, 3, and 4.

Figure 5 presents the comparison between the experimental and simulated electro-optical characteristics of the QD-LED devices. Figures 5a-c show the experimental voltage-dependent characteristics of a CdSe/ZnS core/shell-based red QD-LED device fabricated in this study, including the current density, luminance with an EL photograph inset, and EQE. The CdSe-based QD-LED device exhibits a current density of 0.303 A cm⁻^2^ at 6 V (Fig. 5a), The luminance at 6 V is 23,570 cd m⁻^2^, and the threshold voltage, defined as the voltage at which the luminance reaches 1 cd m^−2^, is 2.0 V (Fig. 5b). The CdSe-based QD-LED exhibits a maximum EQE of 8.65% at 2.4 V, with a EQE droop by 5.6% at 6.0 V after its peak value.

Simulated charge transport characteristics of QD-LED devices using the field-dependent carrier density selection scheme under forward bias voltages ranging from 1 to 6 V, in 0.1 V increments, for different numbers of QD layers, *N*_QD_, are presented in Figs. 5d-i. The maximum EQE is governed by the Langevin radiative recombination constant *B*, the Shockley–Read–Hall non-radiative lifetime *τ*, the Auger non-radiative recombination probability *C*, as well as the optical efficiency of the QD-LED devices. More precisely, the maximum EQE, *η*_max_, is theoretically determined by *η*_max_ = *η*_opt_ × *Q/*(*Q* + 2)^16^, where *η*_opt_ is the optical efficiency of the QD-LED devices, and *Q* is the quality factor defined by *Q* = *B* × (*τ*/*C*)^1/2^. Here, the optical efficiency of the CdSe/ZnS red QD-LED device was measured to be *η*_opt_ = 9.8% in our experiment (Fig. S2). Based on the optical efficiency, the recombination coefficients were carefully tuned so that the simulated maximum EQE matched the experimental values of 8.65% (Table S2). For each applied voltage, a steady-state solution was obtained by time-domain integration of the continuity equations up to 10 μs.

Figure 5d shows the spatial profiles of the valence (red) and conduction (blue) band energy levels across the device structure under the voltage of every 1 V from 1 to 6 V, in 1 V increments, when *N*_QD_ = 2 (see also Fig. S3a for potential distributions and Figs. S3b for space charge density distributions). Different line styles correspond to different applied voltages. At all voltages, a pronounced discontinuity appears in the valence band energy near the HIL/HTL interface, reflecting the valence band offset between the two layers. This discontinuity at *z* = 20.5 nm induces a negative electric field in the valence band, which drives holes to drift from the HTL toward the HIL, as shown in Fig. 5e. This reverse-directional drift is counteracted by a forward-directional hole diffusion current from the HIL to the HTL (Figs. S3c and S3d). The resulting current balance maintains the hole density in the HIL region close to its acceptor doping density under all bias conditions (Fig. 5f). This confirms that, despite the presence of an unavoidable valence band discontinuity, the HIL layer effectively functions as a stable hole-conducting layer in the QD-LED device.

The simulated voltage-dependent device characteristics, including current density, luminance, and EQE, are shown in Figs. 5g-i. Since the turn-on voltage is influenced by the effective thickness of the QD layer, we systematically varied the number of QD layers *N*_QD_ in the simulation (*N*_QD_ = 1, 2, 3, and 4) to examine its effect on current density, luminescence, and EQE characteristics for the applied voltage. In the simulation, when *N*_QD_ = 3, the simulated turn-on voltage closely matched the experimental value of approximately 2 V. Based on this condition, the injection coefficients *S*_p_ and *S*_n_ were further adjusted to 0.46 (Table S2) so that the current density reached 0.3 A cm⁻^2^ at 6 V, in line with the experimental measurement. In the simulation, most material parameters, including carrier mobilities in the transport layers, energy band levels, electrode work functions, and QD structural parameters such as core size and shell thickness (affecting tunnelling probability), were fixed based on experimental values or literature (Tables S1 and S2), while a limited set of parameters was adjusted within physically reasonable ranges. The recombination parameters and effective QD layer thickness were selected to reproduce key physical observables, namely the maximum EQE and threshold voltage, respectively. The injection coefficients were adjusted to match the overall current density level. Importantly, the voltage-dependent trends of current density, luminance, and EQE are predominantly governed by carrier injection processes at the heterojunction interfaces, particularly at the HTL/QD interface, and remain robust against moderate variations of these parameters. As illustrated in Fig. 5g, the current density parabolically increases according to the applied voltage, and as the number of QD layers decreases, the current density abruptly increases. Especially for *N*_QD_ = 3, the current density reached around 0.295 A cm^−2^ at 6.0 V, which is a very similar value compared with the experimental current density in Fig. 5a.

In Fig. 5h, the luminance rises sharply in logarithmic scale, and the luminance increases abruptly as the number of QD layers decreases. The luminance reaches around 20,795 cd m^−2^ at 6.0 V. The threshold voltages for *N*_QD_ = 1, 2, 3, and 4 are observed to be 1.12 V, 1.52 V, 1.86 V, and 2.30 V, respectively. The threshold voltage, *V*_th_, of QD-LEDs is determined by the onset of simultaneous hole and electron injection into the QD layer, enabling radiative recombination defined in Eq. (6) (Methods section). Based on this formulation, the *V*_th_ is approximately predicted by *V*_th_ ≈ (2*N*_QD_/*q*) max(Δ*E*_C0_, Δ*E*_V0_) + *V*_bi_, where *N*_QD_​ is the number of QD layers (proportional to QD thickness), *q* is the elementary charge, Δ*E*_V0_ ​ and Δ*E*_C0_ ​ are the valence and conduction band offsets at the HTL/QD and QD/ETL interfaces, respectively, and *V*_bi_ ​ is the built-in potential determined by the electrode work function difference^12^. In Fig. 5h, increasing *N*_QD_ leads to higher threshold voltages because the reduced electric field across thicker QD stacks requires a larger applied bias to overcome the interfacial band offsets. This confirms that the HTL/QD and QD/ETL interface physics, together with QD thickness, play a dominant role in determining the threshold voltages and overall electro-optical characteristics.

In Fig. 5i, the simulated device exhibits an equal peak EQE of 8.65% at 1.18 V, 1.68 V, 2.26 V, and 2.88 V for the respective number of QD layers, followed by an EQE droop at higher voltages due to Auger recombination^31–33^. Considering the current density, luminescence and EQE curves for the applied voltage, the charge transport simulation model using the field-dependent carrier density scheme shows good agreement with the experimental current density, luminance, and EQE characteristics, closely reproducing their overall trends across the applied voltage. This indicates that the experimental electro-optical properties of the QD-LED devices are accurately captured by the charge transport simulation model combined with the electric-field-dependent carrier density selection scheme at the HIL/HTL interface.

To further quantitatively compare the simulation and experimental results, the current density, luminance, and EQE characteristics are presented in Fig. S4. Because the simulated threshold voltage is approximately 0.14 V lower than the experimental value, the simulated curves are slightly shifted along the voltage axis to enable a voltage-aligned comparison that focuses on the voltage-dependent behaviour. The comparison is evaluated using the root-mean-square error (RMSE) and mean absolute percentage deviation (PD), as defined in Supplementary Information. Over the 2.0–6.0 V range, the RMSE values are 34.70 mA cm^−2^ for current density, 0.388 for log_10_ (luminance), and 0.479% for EQE, with corresponding percentage deviations of 61.83%, 55.57%, and 8.13%, respectively. These results indicate that the model reproduces the overall voltage-dependent trends and the shapes of the electro-optical characteristics with reasonable quantitative consistency, while a residual threshold-voltage offset remains. Since the threshold voltage is primarily determined by carrier injection into the QD layer and is governed by the band offsets at the HTL/QD and QD/ETL interfaces, this offset is attributed to uncertainties in these interfacial energy alignments. Small variations in the effective band offsets, along with related material parameters, can therefore lead to a noticeable shift in the predicted turn-on voltage. While the parameter values were selected within physically reasonable ranges and only minimally adjusted for scale alignment, the agreement should be interpreted in terms of trend-level consistency rather than exact quantitative matching, as fully parameter-free validation would require independently measured material parameters for each layer.

**Fig. 6:**
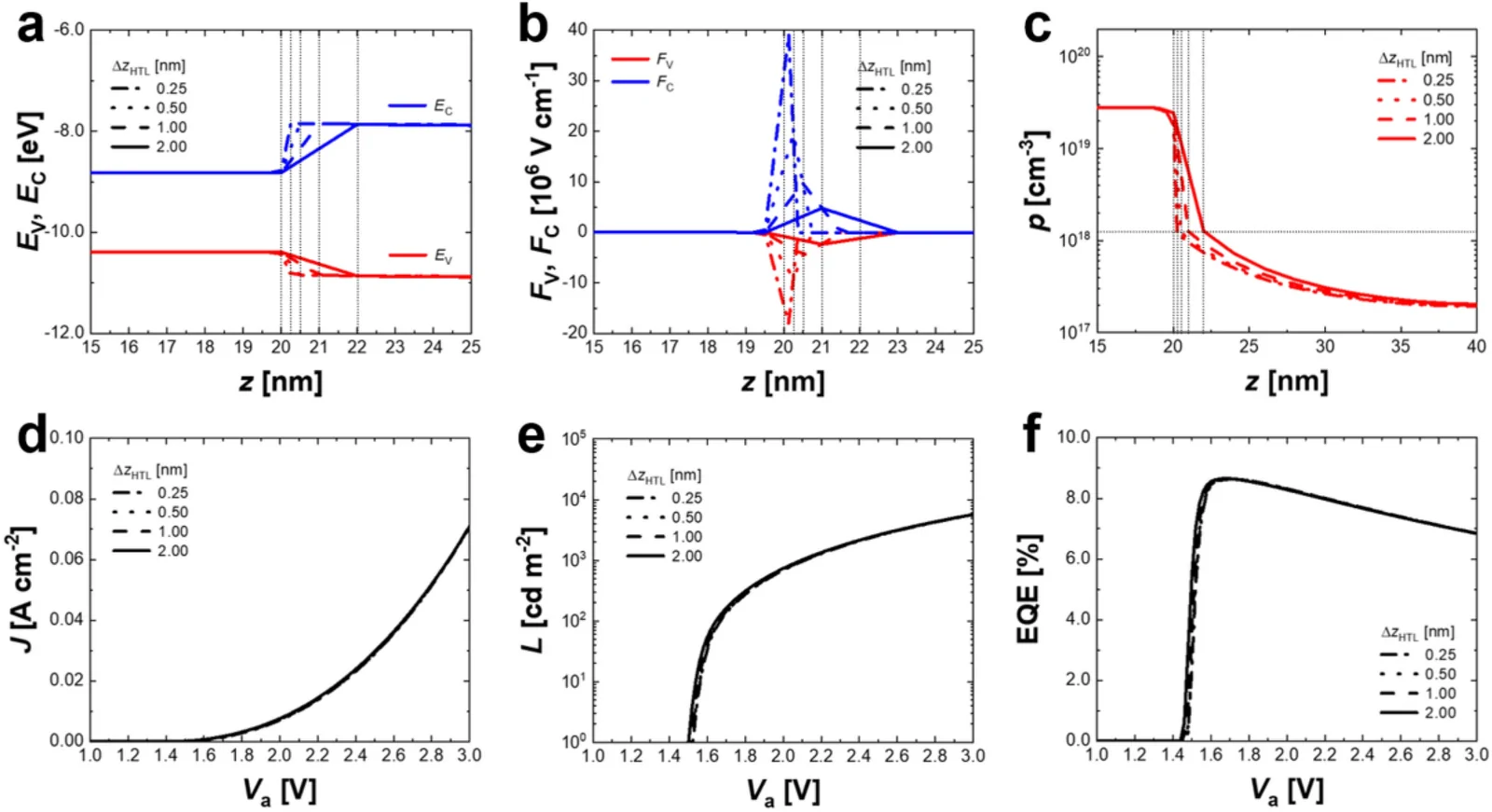
Impact of grid spacing ​Δ*z*_HTL_ in the HTL on charge transport and electro-optical simulation results of QD-LED device using an electric-field-dependent carrier density selection scheme. (**a**) Energy band profiles, (**b**) electric-field distribution, and (**c**) hole density distribution near the HIL/HTL interface at the applied voltage of 6 V for different grid spacings ​Δ*z*_HTL_ = 0.25 nm, 0.5 nm, 1.0 nm, and 2.0 nm. Voltage-dependent electro-optical characteristics of (**d**) current density, (**e**) luminance, and (f) EQE, showing minor variations with grid spacing.

Figure 6 presents the impact of grid spacing in the HTL on the simulated electro-optical characteristics in QD-LED devices. Grid spacings Δ*z*_HTL_ of 0.25 nm, 0.5 nm, 1.0 nm, and 2.0 nm were examined, corresponding to 160, 80, 40, and 20 spatial grids, respectively, across the 40 nm-thick HTL. Figures 6a-c illustrate the charge transport behaviour near the HIL/HTL interface on an enlarged scale (full-scale plots across the entire device are provided in Fig. S5). As illustrated in Fig. 6a, the slopes of the valence and conduction band energy levels across the HIL/HTL interface become progressively less steep with increasing grid spacing (also see Fig. S5a), because the potential across the heterojunction is barely affected by the grid spacing (Fig. S5b). As a consequence, the electric field experienced by holes in the valence band becomes weaker (i.e., less negative) as Δ*z*_HTL_ increases (Fig. 6b and Fig. S5c). Similarly, the electric field experienced by electrons in the conduction band decreases in the positive direction, consistent with the conduction band alignment at the interface. As the hole diffusion current must balance the drift current induced by the electric fields, the gradient in hole density also becomes less steep with increasing grid spacing (Fig. 6c and Fig. S5d).

Figures 6d-f show the simulated current density, luminance, and EQE as functions of the applied voltage for different grid spacings. As shown in Fig. 6d, the current density–voltage characteristics remain largely unchanged across the tested grid spacings. The luminance and EQE curves (Figs. 6e and f) exhibit slight shifts toward higher voltages as the grid spacing decreases, with threshold voltage variations of less than 0.03 V. However, finer grid spacings result in significantly increased computational time due to the larger number of nodal points required to cover the same HTL thickness (Fig. S6). These results indicate that the choice of grid spacing has only a limited influence on the simulated electro-optical performance when the field-dependent carrier density selection scheme is used at the HIL/HTL interface. Therefore, a moderate grid spacing of approximately 1 nm provides a good balance between computational efficiency and numerical accuracy in QD-LED device simulations. Following the grid-spacing analysis, we further investigated numerical stability through a linearised stability analysis and direct numerical tests (see Methods and Table S3). Stable convergence was observed for (*µ*_p_^HTL^*k*_B_*T/q*)Δ*t/*Δz^2^ < 0.083, which is significantly more restrictive than the classical stability limit (< 0.5) due to the additional stiffness arising from drift transport, nonlinear recombination, and abrupt heterojunction discontinuities.

### Impact of HIL properties on the electro-optical characteristics of QD-LED devices

**Fig. 7:**
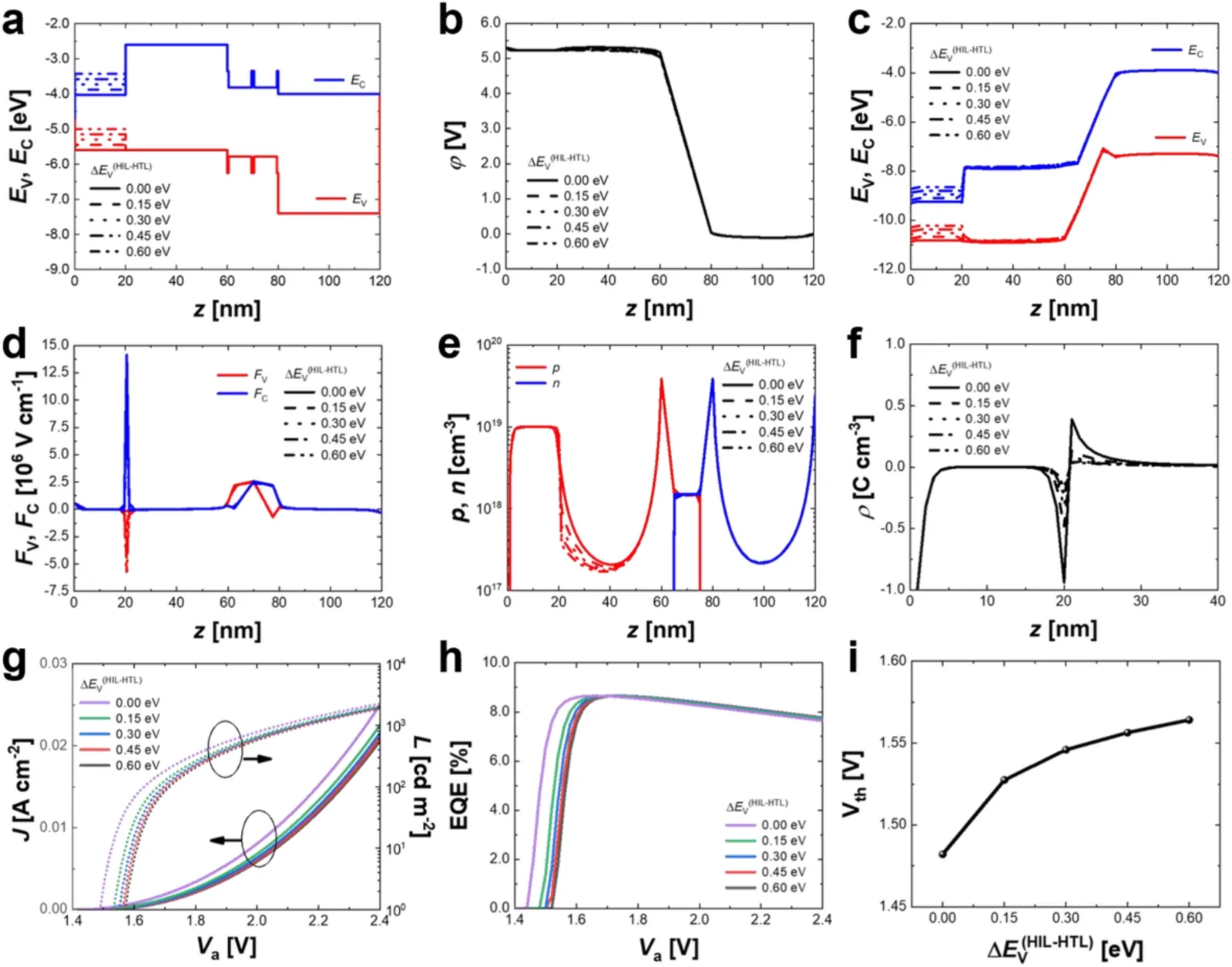
Charge transport simulation results for QD-LED devices with different valence band energy offsets Δ*E*_V_^(HIL-HTL)^ at the HIL/HTL interface: 0.00 eV, 0.15 eV, 0.30 eV, 0.45 eV, and 0.60 eV. (**a**) Flat-band energy level configurations, simulated (**b**) potential distributions, (**c**) valence and conduction band energy profiles, (**d**) electric field distributions in the valence and conduction bands, (**e**) hole and electron density distributions, and (**f**) charge density distribution near the HIL/HTL heterojunction. Simulated voltage-dependent (**g**) current density and luminance and (**h**) EQE characteristics of QD-LED devices. (**i**) Threshold voltage *V*_th_ as a function of valence band energy offset, Δ*E*_V_^(HIL-HTL)^.

Figure 7 illustrates the effect of valence band energy alignment at the HIL/HTL interface on the charge transport and electro-optical performance of QD-LED devices. To examine the influence of energy level alignment, the valence band offset Δ*E*^V(HIL-HTL)^ was varied from 0.0 eV to 0.6 eV in 0.15 eV increments while keeping the bandgap of the HIL constant. Accordingly, both the valence and conduction bands of the HIL were simultaneously shifted upward as the offset increased (Fig. 7a). As shown in Fig. 7b and Fig. S7a, the potential in the HTL region decreases with increasing Δ*E*^V(HIL-HTL)^, resulting in a slightly upward shift of both valence and conduction band energy levels in the HTL (Fig. 7c). Since the electric field at the HIL/HTL interface is strongly influenced by the band offset, a smaller offset produces a weaker electric field at *z* = 20.5 nm in the valence band (Fig. 7d and Fig. S7b), thereby reducing the hole drift current density from HTL to HIL across the interface. Due to the reduced hole drift in the negative direction, more holes are diffused to the HTL layer from the HIL, resulting in a gentler gradient in hole density (Fig. 7e). This leads to wider charge accumulation regions in the HTL around the interface, with charge depletion on the HIL side (Fig. 7f and Fig. S7c).

The energy level alignment at the HIL/HTL interface directly affects the overall device performance. Figure 7g shows the current density and luminance curves according to the applied voltage for the different valence band offset Δ*E*^V(HIL-HTL)^ from 0.0 eV to 0.6 eV in 0.15 eV increments. As shown in Fig. 7g, devices with smaller valence band offsets exhibit more rapid increases in current density and luminance with applied voltage, due to the higher electric field within the EML caused by the increase of potential at the HTL (Figs. S7a and d). The luminance curves shift toward lower voltages as the HIL/HTL band offset decreases due to the enhanced electric field at the EML for the easier carrier injection process. The EQE curves also shift toward lower voltages as the HIL/HTL band offset decreases, resulting in a reduced operating voltage and enhanced EQEs within the low voltage range (Fig. 7h).

In Fig. 7i, the threshold voltage shifts from 1.48 V to 1.56 V as the band offset increases from 0.0 eV to 0.6 eV in our simulation, demonstrating its sensitivity to the interfacial band offset. As the voltage increases, the threshold voltage initially exhibits a large increase when the energy level rises from 0.0 eV to 0.15 eV. Beyond this, however, the slope of the threshold voltage progressively decreases as the valence band offset increases. These results confirm that the energy alignment at the HIL/HTL heterojunction plays a critical role in determining carrier injection to the QD layer and overall device performance. Accordingly, careful engineering of the HIL energy levels, particularly the valence band offset, is one of the essential strategies for achieving charge balance between hole and electron injection, thereby enabling high-performance QD-LED devices.

**Fig. 8:**
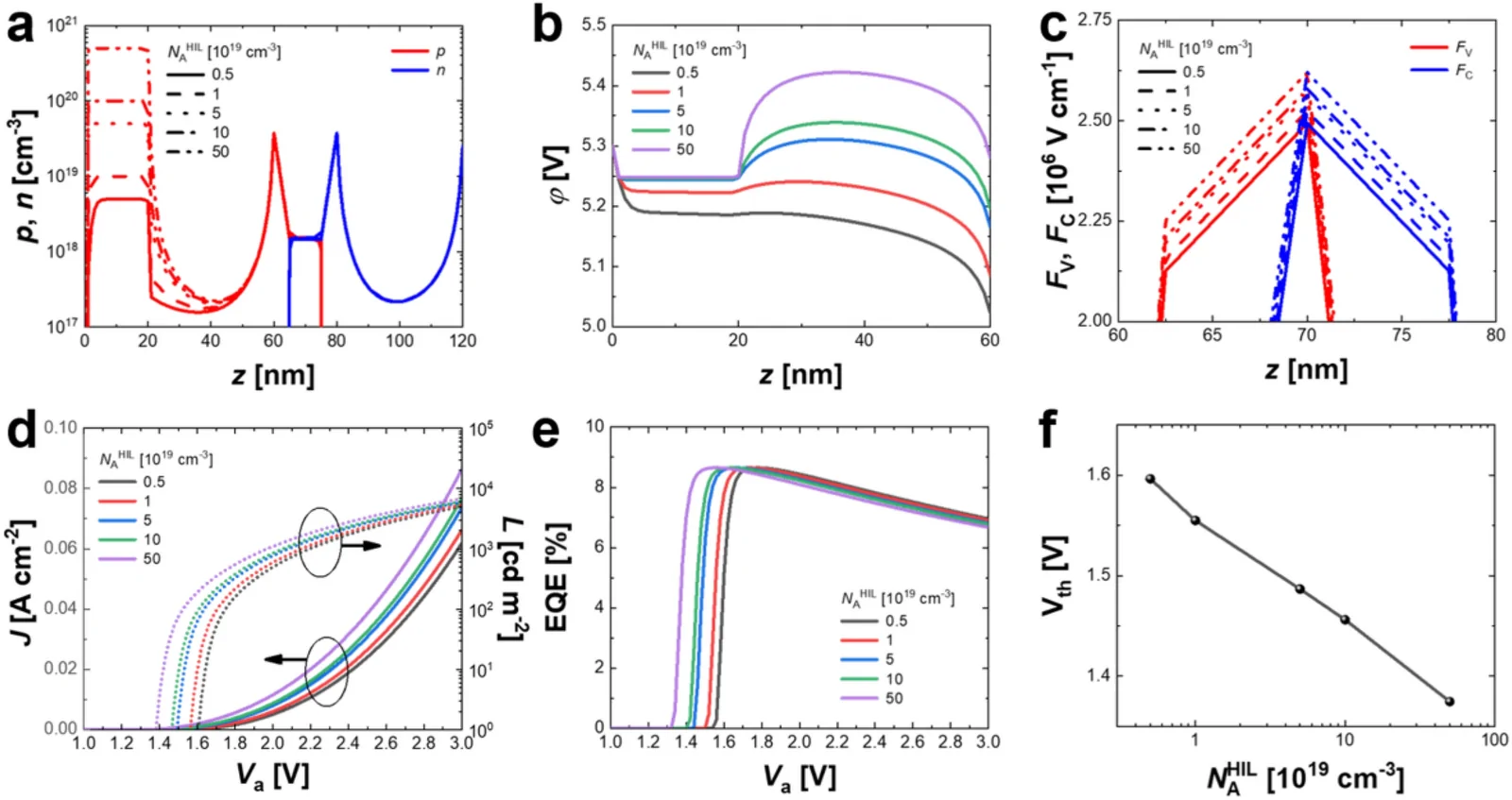
Charge transport simulation results for QD-LED devices with varying hole doping densities in the HIL layer: *N*_A_^HIL^ = 0.5 × 10^19^ cm^−3^, 1.0 × 10^19^ cm^−3^, 5.0 × 10^19^ cm^−3^, 10.0 × 10^19^ cm^−3^, and 50.0 × 10^19^ cm^−3^. (**a**) Valence and conduction band energy profiles, (**b**) electric field distributions in the valence and conduction bands, and (**c**) hole and electron density distributions across the QD-LED device for different *N*_A_^HIL^. Simulated voltage-dependent (**d**) current density and luminance and (**e**) EQE characteristics of QD-LED devices. (**f**) Threshold voltage *V*_th_ as a function of hole doping density in the HIL, *N*_A_^HIL^.

Figure 8 illustrates the dependence of the electro-optical characteristics of the QD-LED devices on the acceptor doping density in the HIL, denoted as *N*_A_^HIL^. The doping densities are set to be 0.5 × 10^19^ cm^−3^, 1.0 × 10^19^ cm^−3^, 5.0 × 10^19^ cm^−3^, 1.0 × 10^20^ cm^−3^, and 5.0 × 10^20^ cm^−3^. As the acceptor doping density in the HIL increases, a greater number of holes diffuse from the HIL into the HTL across the HIL/HTL interface, leading to an expanded hole accumulation region on the HTL side (Fig. 8a and Fig. S8a). This accumulation of positive carriers in the HTL results in an increase in the electrostatic potential within HTL (Fig. 8b and Figs. S8b and c). Since the electric fields in the EML are determined by the potential difference between the HTL and ETL boundaries facing the EML, the electric fields for the hole and electron become stronger as the HIL acceptor doping density increases (Fig. 8c and Fig. S8d).

This enhanced carrier supply from the HIL to HTL facilitates stronger electric fields across the EML and enables more efficient carrier injection due to the electric-field-dependent carrier capture process, resulting in increased current density and luminance at a given applied voltage (Fig. 8d). Accordingly, the corresponding EQE curves shift toward lower voltages as the acceptor doping density in the HIL increases (Fig. 8e). The threshold voltage decreases linearly from 1.60 V to 1.37 V as the acceptor doping density increases logarithmically from 0.5 × 10^19^ cm^−3^ to 5.0 × 10^20^ cm^−3^, demonstrating its sensitivity to the HIL doping density. This trend indicates that enhanced carrier injection, enabled by higher HIL doping levels, improves the luminance and EQE of the QD-LED device at a given applied voltage, thereby reducing the operation voltage and enhancing overall electro-optical efficiency. Therefore, optimising the HIL doping concentration also represents an effective strategy for improving the performance of the QD-LED devices.

## Discussion

In this study, we developed an advanced numerical charge transport model to describe carrier dynamics across the heterojunction interface between the HIL and the HTL in QD-LED devices. The HIL/HTL heterojunction interface is characterised by discontinuities in energy band alignment and doping concentration. To accurately model the drift current density across this interface, a field-dependent carrier density selection scheme was introduced in the numerical discretisation. The numerical model was validated using an HOD with an anode/HIL/HTL/cathode bilayer structure isolated from the full QD-LED stack, and further validated experimentally with the CdSe/ZnS-based red QD-LED device. This modelling framework successfully reproduced typical voltage-dependent current density, luminance, and EQE characteristics, demonstrating good agreement with experimental data and accurately capturing the essential charge transport behaviour of QD-LEDs. Using this model, we evaluated the influence of spatial grid spacing in the HTL and material properties of the HIL on device performance. The simulation results confirmed that grid spacing in the HTL has only a limited impact on electro-optical characteristics, validating the numerical stability and computational efficiency of the model.

Furthermore, systematic analysis of HIL material properties showed that minimising the valence band offset at the HIL/HTL interface and increasing the acceptor doping density in the HIL enhanced charge carrier injection to the EML, resulting in reduced operating voltage and improved luminance, thereby contributing to the overall electro-optical efficiency of QD-LED devices within a low voltage range. Notably, the present model provides a quantitative understanding of how band alignment and doping density control interfacial charge accumulation, potential, and electric field. This offers a self-consistent explanation of carrier injection behaviour that was previously understood mainly qualitatively.

The proposed approach is valid for describing carrier transport across heterojunction interfaces where abrupt variations in energy band alignment and doping density can be treated within a drift–diffusion framework. In this work, the model is applied to organic semiconductor layers such as PEDOT:PSS and TFB, where drift–diffusion provides a reasonable approximation of charge transport. However, the present model does not explicitly account for additional physical effects relevant to organic semiconductors, such as energetic disorder and other interfacial effects, which may influence charge transport across the HIL/HTL interface. Extending the model to include energetic disorder would require modifications to the carrier transport formulation to account for the distributed density of states and field-dependent hopping processes. Incorporating these effects into an extended drift–diffusion framework could further improve the physical accuracy of the model in future studies. In conclusion, the proposed numerical model provides a reliable and physically consistent computational framework for analysing and optimising heterojunction interfaces in QD-LEDs and is expected to support the optimal design of high-performance, energy-efficient, next-generation displays based on QD-LED technology.

## Methods

### Charge transport model in QD-LED device

Assuming a homogeneous and infinitely extended device plane along the *xy*-plane, the charge carrier transport of holes and electrons is mathematically formulated by the one-dimensional continuity equations at a given position *z* along the *z*-axis and time *t*, as presented in Eq. (5).

5 $$\begin{gathered} \frac{{\partial p\left({z,t} \right)}}{\partial t} = - \frac{1}{q}\frac{{\partial J_{\mathrm{p}} \left( {z,t} \right)}}{\partial z} - U\left( {z,t} \right), \hfill \\ \frac{{\partial n\left( {z,t} \right)}}{\partial t} = \frac{1}{q}\frac{{\partial J_{\mathrm{n}} \left( {z,t} \right)}}{\partial z} - U\left( {z,t} \right). \hfill \\ \end{gathered}$$

Here, *p*(*z*,* t*) and *n*(*z*,* t*) represent the hole and electron densities at a given device location *z* and time *t*. *q* is the proton charge, *J*_p_(*z*,*t*) and *J*_n_(*z*,*t*) are the electric current density of holes and electrons, and *U*(*z,t*) is the recombination rate of the carriers. The current density is divided into the injection, hopping, and drift–diffusion current densities according to the device locations.

First, the injection current densities *J*_p_^I^ and *J*_n_^I^ associated with the electric-field-dependent charge capture process between the QD layer and transport layers are described by Eq. (6)^12^.

6 $$\begin{gathered} J_{\mathrm{p}}^{\mathrm{I}} = qr_{\mathrm{QD}} \left( {\pi r_{\mathrm{QD}}^{2} } \right)S_{\mathrm{p}} T_{\mathrm{bp}} \mu_{\mathrm{p}}^{\mathrm{QD}} F_{\mathrm{VH}} p_{\mathrm{H}} \left( {M_{\mathrm{QD}} - p_{\mathrm{Q,1}} } \right), \hfill \\ J_{\mathrm{n}}^{\mathrm{I}} = qr_{\mathrm{QD}} \left( {\pi r_{\mathrm{QD}}^{2} } \right)S_{\mathrm{n}} T_{\mathrm{bn}} \mu_{\mathrm{n}}^{\mathrm{QD}} F_{\mathrm{CE}} n_{\mathrm{E}} \left( {M_{\mathrm{QD}} - n_{\mathrm{Q},M} } \right). \hfill \\ \end{gathered}$$

Here, *r*_*QD*_ is the radius of QD, and *S*_p_ and *S*_n_ are charge injection coefficients for the hole and electron, respectively. *T*_bp_ and *T*_bn_ are tunnelling probabilities of the energy barriers for the hole and electron formed by the shell of QDs^12^. *μ*_p_^QD^ and *μ*_n_^QD^ are the hole and electron mobilities of the QD layer, where *μ*_p_^QD^ = *μ*_p0_^QD^(*F*_V_/*F*_0_)^1/2^ and *μ*_n_^QD^ = *μ*_n0_^QD^(*F*_C_/*F*_0_)^1/2^ for reference hole and electron mobilities *μ*_p0_^QD^ and *μ*_n0_^QD^ at a given electric field *F*_0_, according to the Poole–Frenkel law^5^. *F*_VH_ and *F*_CE_ are the electric fields at the HTL/EML and EML/ETL interfaces, respectively. *p*_H_ and *n*_E_ are the hole and electron densities at the HTL and ETL surfaces facing the QD layer, respectively. *M*_QD_ is the density of QDs obtained by the reciprocal of the volume of a single QD particle. *p*_Q,1_ and *n*_Q,*M*_ are the hole and electron densities at the centre of the outermost QD layers facing HTL and ETL, respectively.

Next, the hopping current densities of the hole and electron between neighbouring QDs, *J*_p_^H^ and *J*_n_^H^, are described by the carrier exchanges through the quantum tunnelling barrier between two adjacent QDs as described in Eq. (7)^12,15,16^.

7 $$\begin{gathered} J_{\mathrm{p}}^{\mathrm{H}} = - q\nu_{\mathrm{p}} d_{\mathrm{QD}} \left( {p_{g} - p_{g + 1} } \right), \hfill \\ J_{\mathrm{n}}^{\mathrm{H}} = q\nu_{\mathrm{n}} d_{\mathrm{QD}} \left( {n_{g} - n_{g + 1} } \right). \hfill \\ \end{gathered}$$

Here, *ν*_p_ and *ν*_n_ are tunnelling frequencies^12^ of the hole and electron oscillating between two QDs indexed with the natural number *g* and *g* + 1, where 0 ≤ *g* ≤ *N*_QD_ – 1.

Finally, the drift–diffusion current densities, *J*_p_^D^ and *J*_n_^D^, are described by the sum of the drift and diffusion current densities as expressed in Eq. (8)^12,15,16^.

8 $$\begin{gathered} J_{\mathrm{p}}^{\mathrm{D}} = q\mu_{\mathrm{p}} pF_{\mathrm{V}} - \mu_{\mathrm{p}} k_{\mathrm{B}} T\frac{\partial p}{{\partial z}} , \hfill \\ J_{\mathrm{n}}^{\mathrm{D}} = q\mu_{\mathrm{n}} nF_{\mathrm{C}} + \mu_{\mathrm{n}} k_{\mathrm{B}} T\frac{\partial n}{{\partial z}} . \hfill \\ \end{gathered}$$

Here, *μ*_p_ and *μ*_n_ are the hole and electron mobilities in the given semiconductor layers, and *F*_V_ and *F*_C_ are the spatial electric field intensities acting on the hole and electron. *k*_B_ is the Boltzmann constant, and *T* is the absolute temperature of the device.

The recombination rate *U* in Eq. (5) is defined by the sum of the SRH recombination rate *U*_SRH_, the Auger recombination rate *U*_AUG_, and the Langevin radiative recombination rate *U*_RAD_. As non-radiative recombination rates, the SRH and Auger recombination rates are expressed as Eqs. (9) and (10), respectively^1,34^.

9 $$U_{{{\mathrm{SRH}}}} = \frac{{pn - n_{{\mathrm{i}}}^{2} }}{{\tau_{{\mathrm{n}}} \left( {p + n_{{\mathrm{i}}} } \right) + \tau_{{\mathrm{p}}} \left( {n + n_{{\mathrm{i}}} } \right)}}$$

10 $$U_{{{\mathrm{AUG}}}} = \left( {C_{{\mathrm{p}}} p + C_{{\mathrm{n}}} n} \right)\left( {pn - n_{{\mathrm{i}}}^{2} } \right)$$

Here,* n*_i_ is the intrinsic carrier density of the given material. *τ*_p_ and *τ*_n_ are the hole and electron SRH recombination lifetimes, and *C*_p_ and *C*_n_ are the hole and electron Auger capture probabilities, respectively. The radiative recombination at the centre of the QDs with the index *g* in the EML layer is expressed as Eq. (11).

11 $$U_{{{\mathrm{RAD}}}} = \gamma \left( {p_{g} n_{g} - n_{{{\mathrm{i}},g}}^{2} } \right)$$

Here, *γ* is the Langevin recombination strength, which is calculated from *γ* = *q*(*μ*_p0_^QD^ + *μ*_n0_^QD^)/(*ε*_r_*ε*_0_) with the dielectric constant, *ε*_r_, of a QD layer and the permittivity of free space, *ε*_0_^5^. The material parameters for QDs used in the simulation are listed in Table S2.

The electric field intensity *F*_V_ and *F*_C_ are calculated by the derivatives of conduction and valence energy levels, such that *F*_V_ = (1/*q*)∂*E*_V_(*z*)/∂*z* and *F*_C_ = (1/*q*)∂*E*_C_(*z*)/∂*z*, where the *E*_V_(*z*) = *E*_V0_(*z*) ‒ *qφ*(*z*) and *E*_C_(*z*) = *E*_C0_(*z*) ‒ *qφ*(*z*), respectively, from the electric potential *φ*(*z*) across the device. *E*_C0_(*z*) and *E*_V0_(*z*) are the discontinuous flat-band energy-level distributions of valence and conduction bands, which are plotted in Fig. 1b. The electrostatic potential *φ*(*z*) is determined by the following Poisson’s Eq. 12.

12 $$\varepsilon_{0} \frac{\partial }{\partial z}\left( {\varepsilon_{{\mathrm{r}}} \frac{\partial \varphi }{{\partial z}}} \right) = - q\left( {p - n + N_{{\mathrm{D}}} - N_{{\mathrm{A}}} } \right)$$

Here, *ε*_0_ and *ε*_r_ are the permittivity of free space and the dielectric constant of a given material, respectively. *N*_D_ and *N*_A_ are the donor and acceptor doping densities of semiconductor layers. With the given current densities defined in each region of the device, the dynamic motion of the charge carriers is calculated by solving the continuity equations and Poisson’s equation simultaneously.

### Discretisation of current densities across QD-LED devices

First, the drift–diffusion current densities *J*_p,*l*+1/2_^* k*^, *J*_p,*l*−1/2_^* k*^, *J*_n,*l*+1/2_^* k*^, and *J*_n,*l*−1/2_^* k*^ for hole and electron at the *k*-th time step on *l* + 1/2 and *l*−1/2 nodes are discretised by Eqs. (13) and (14).

13 $$\begin{gathered} J_{\mathrm{p},l + 1/2}^{\mathrm{D}, k} = q\mu_{\mathrm{p}} p_{l + 1/2}^{k} F_{\mathrm{V},l + 1/2}^{k} - \mu_{\mathrm{p}} k_{\mathrm{B}} T\frac{{p_{l + 1}^{k} - p_{l}^{k} }}{{z_{l + 1} - z_{l} }} , \hfill \\ J_{\mathrm{p},l - 1/2}^{\mathrm{D},k} = q\mu_{\mathrm{p}} p_{l - 1/2}^{k} F_{\mathrm{V},l - 1/2}^{k} - \mu_{\mathrm{p}} k_{\mathrm{B}} T\frac{{p_{l}^{k} - p_{l - 1}^{k} }}{{z_{l} - z_{l - 1} }} . \hfill \\ \end{gathered}$$

14 $$\begin{gathered} J_{\mathrm{n},l + 1/2}^{\mathrm{D}, k} = q\mu_{\mathrm{n}} n_{l + 1/2}^{k} F_{\mathrm{C},l + 1/2}^{k} + \mu_{\mathrm{n}} k_{\mathrm{B}} T\frac{{n_{l + 1}^{k} - n_{l}^{k} }}{{z_{l + 1} - z_{l} }} , \hfill \\ J_{\mathrm{n},l - 1/2}^{\mathrm{D}, k} = q\mu_{\mathrm{n}} n_{l - 1/2}^{k} F_{\mathrm{C},l - 1/2}^{k} + \mu_{\mathrm{n}} k_{\mathrm{B}} T\frac{{n_{l}^{k} - n_{l - 1}^{k} }}{{z_{l} - z_{l - 1} }}. \hfill \\ \end{gathered}$$

Here, *p*_*l*+1/2_^* k*^, *p*_*l*−1/2_^* k*^, *n*_*l*+1/2_^* k*^, and *n*_*l*−1/2_^* k*^ are described by *p*_*l*+1/2_^* k*^ = (*p*_*l*+1_^* k*^ + *p*_*l*_)/2, *p*_*l*−1/2_^* k*^ = (*p*_*l*_^*k*^ + *p*_*l*−1_^* k*^)/2, *n*_*l*+1/2_^* k*^ = (*n*_*l*+1_^* k*^ + *n*_*l*_^*k*^)/2, and *n*_*l*−1/2_^* k*^ = (*n*_*l*_^*k*^ + *n*_*l*−1_^* k*^)/2, respectively, except when *l* = HI for *p*_HI+1/2_^* k*^ and *n*_HI+1/2_^* k*^.

The hole and electron injection current densities are discretised by Eq. (15) according to the numerical grid space defined in Fig. 2.

15 $$\begin{gathered} J_{{\mathrm{p,H+1/2}}}^{\mathrm{I},k} = qr_{\mathrm{QD}} \left( {\pi r_{\mathrm{QD}}^{2} } \right)S_{\mathrm{p}} T_{\mathrm{bp}} \mu_{\mathrm{p}}^{\mathrm{QD}} F_{\text{V, H+1/2}}^{k} p_{\mathrm{H}}^{k} \left( {M_{QD} - p_{\mathrm{H+1}}^{k} } \right), \hfill \\ J_{\mathrm{n,E-1/2}}^{\mathrm{I},k} = qr_{\mathrm{QD}} \left( {\pi r_{\mathrm{QD}}^{2} } \right)S_{\mathrm{n}} T_{\mathrm{bn}} \mu_{n}^{QD} F_{\mathrm{C,E-1/2}}^{k} n_{\mathrm{E}}^{k} \left( {M_{\mathrm{QD}} - n_{\mathrm{E-1}}^{k} } \right). \hfill \\ \end{gathered}$$

Here, H and E denote the nodal indices corresponding to the right boundary of HTL and the left boundary of ETL, respectively, both adjacent to the QD layers.

The carrier hopping current density defined between two adjacent QDs indexed as *g* and *g* + 1 is discretised by Eq. (16).

16 $$\begin{gathered} J_{{\mathrm{p},{g + 1/2}}}^{\mathrm{H},k} = - q\nu_{\mathrm{p}} d_{\mathrm{QD}} \left( {p_{g}^{k} - p_{g + 1}^{k} } \right), \hfill \\ J_{{\mathrm{n},{g+1/2}}}^{\mathrm{H},k} = q\nu_{\mathrm{n}} d_{\mathrm{QD}} \left( {n_{g}^{k} - n_{g + 1}^{k} } \right). \hfill \\ \end{gathered}$$

### Discretisation of poisson’s equation

Among the current densities in the continuity equation, all current components involved in carrier drift or injection are coupled with the electric field across the entire device, which is determined by Poisson’s equation. The given Poisson equation is also discretised using the FDM and formulated as Eq. (17).

17 $$\frac{2}{{z_{l + 1} - z_{l - 1} }}\left( {\varepsilon_{\mathrm{r},l + 1/2} \left. {\frac{\partial \varphi }{{\partial z}}} \right|_{l + 1/2} - \varepsilon_{\mathrm{r},l - 1/2} \left. {\frac{\partial \varphi }{{\partial z}}} \right|_{l - 1/2} } \right) = - \frac{q}{{\varepsilon_{0} }}\left( {p_{l} - n_{l} + N_{{{\mathrm{D}},l}} - N_{{{\mathrm{A}},l}} } \right)$$

Here, each term inside the brackets on the left-hand side represents the electric displacement at the central positions between nodes *l* and *l* + 1 (*l* + 1/2) and between *l* and *l*−1 (*l*−1/2), respectively. The right-hand side denotes the spatial charge density at the *l*-th node. The electric displacement at each central node is discretised in the calculation grid system as expressed in Eq. (18).

18 $$\begin{gathered} \varepsilon_{\mathrm{r},l + 1/2} \left. {\frac{\partial \varphi }{{\partial z}}} \right|_{l + 1/2} = \frac{{\varepsilon_{\mathrm{r},l + 1} + \varepsilon_{\mathrm{r},l} }}{{2\left( {z_{l + 1} - z_{l} } \right)}}\left( {\varphi_{l + 1} - \varphi_{l} } \right) \hfill \\ \varepsilon_{\mathrm{r},l - 1/2} \left. {\frac{\partial \varphi }{{\partial z}}} \right|_{l - 1/2} = \frac{{\varepsilon_{\mathrm{r},l - 1} + \varepsilon_{\mathrm{r},l} }}{{2\left( {z_{l} - z_{l - 1} } \right)}}\left( {\varphi_{l} - \varphi_{l - 1} } \right) \hfill \\ \end{gathered}$$

As a result, for the *L*−1 number of unknowns *φ*_*l*_ defined at *l* = 1, 2, …, *L* – 1, a system of linear equations as expressed in Eq. (19) is obtained.

19 $$a_{l} \varphi_{l - 1} + b_{l} \varphi_{l} + c_{l} \varphi_{l + 1} = d_{l}$$

When listing these linear equations for *l*, they form a tridiagonal linear matrix equation **Aφ** = **S** for the *L* – 1 unknowns *φ*_*l*_, where **A** and **S** represent the coefficient matrix and the source vector, respectively. **φ** = [*φ*_1_, *φ*_2_, *φ*_3_, …, *φ*_*L*−1_]^T^ is the unknown vector for the potential field across the device, with the superscript T for the transpose of the vector. In this study, the tridiagonal linear matrix system is solved iteratively using the column approximate minimum degree ordering method in the Eigen library^35,36^ to obtain a solution based on the given spatial charge density and the boundary conditions in the computational domain.

### Linearised stability analysis

Our continuity equation with drift–diffusion current can be simplified by the reaction–diffusion equation with diffusion coefficient *α* and reaction coefficient *β*, as described in Eq. (20),

20 $$\frac{\partial u}{{\partial t}} = \alpha \frac{{\partial^{2} u}}{{\partial z^{2} }} + \beta u$$

discretised using the same explicit forward-time central-space (FTCS) scheme as employed in our model. For the given reaction–diffusion equation, a standard von Neumann analysis yields the stability condition

21 $$C = \frac{\alpha \Delta t}{{\Delta z^{2} }} \le \frac{2 + \beta \Delta t}{4}, \beta \le 0$$

for the given time and space step Δ*t* and Δz, respectively^37^. This recovers the classical diffusion stabilty limit *C* = αΔ*t*/Δz^2^ ≤ 1/2 when *β* = 0, and indicates a more restrictive condition in the presence of a negative reaction term.

### Direct numerical stability test

We also systematically evaluated convergence behaviour across a range of Δ*t* and Δ*z*_HTL_ (grid size in HTL region). Table S3 presents a direct numerical stability test of the explicit time integration scheme by varying the temporal and spatial discretisation parameters. The stability behaviour is characterised using the dimensionless parameter *C* = *α*Δ*t*/Δz^2^, which combines the effects of the time step and grid spacing. Here, *α* is defined by *α* = *µ*_p_^HTL^*k*_B_*T*/*q* with the hole mobility *µ*_p_^HTL^ = 2.0 × 10^–3^ cm^2^ V^−1^ s^−1^ at HTL region (Table S1). The simulation results are assessed using the total current density across the device, *J*, at a driving voltage of 5 V after 10 µs. The results show that stable convergence is achieved when *C* ≤ 0.083, whereas larger values lead to numerical divergence. This threshold is significantly more restrictive than the classical diffusion limit (*C* < 0.5), reflecting the additional stiffness introduced by drift transport, nonlinear recombination, and abrupt heterojunction discontinuities in the present model.

### Fabrication and characterisation of QD-LED devices

Red QD-LED devices were fabricated with a conventional device structure of ITO/PEDOT:PSS/TFB/QDs/ZnO/Al. Patterned ITO-coated glass substrates (100 nm thickness, Ossila Ltd.) were sequentially cleaned in acetone and isopropyl alcohol (IPA) via ultrasonication, followed by oxygen plasma treatment. A HIL (PEDOT:PSS, AI 4083, Ossila Ltd.) was spin-coated at 4,000 revolutions per minute (rpm) for 60 s and annealed at 150 °C for 10 min. Subsequently, an HTL (TFB, 12 mg mL⁻^1^ in chlorobenzene, American Dye Sources) was deposited by spin-coating at 3000 rpm for 60 s and annealed at 150 °C for 30 min. CdSe/ZnS core/shell QDs with a peak wavelength of 625 nm (12.5 mg mL⁻^1^ in octane, Suzhou Xingshuo Nanotech Co., Ltd.) were used as the emissive layer and spin-coated at 3,000 rpm, followed by annealing at 100 °C for 10 min. The electron transport layer, composed of ZnO nanocrystals (25 mg mL^−1^ in butanol), was spin-coated at 2,000 rpm and thermally treated at 100 °C for 10 min. Finally, an Al cathode (Testbourne Ltd.) was coated by a thermal evaporation process under high vacuum. The devices, with an active area of 3.0 mm × 1.5 mm, were encapsulated inside a nitrogen-filled glovebox using UV-curable epoxy (Thorlabs Ltd.). Current density, luminance, and external quantum efficiency (EQE) were characterised using an EQE measurement system (C9920-12, Hamamatsu Photonics K.K.).

## Supplementary Information


Supplementary Information.


## Data Availability

The data that support the plots within this paper and other findings of this study are available from the corresponding author upon reasonable request.

## Code availability

The codes developed in this study are available from the corresponding author upon reasonable request.
