# Supplementary material for: Computational carrier dynamics across heterojunction interface between hole injection and transport layers in quantum-dot light-emitting diodes
Source: Sci Rep. 2026 Jun 16;16:27446. doi: 10.1038/s41598-026-55391-2 (PMC13534670; doi:10.1038/s41598-026-55391-2)
Supplement: Supplementary file 1 [file 41598_2026_55391_MOESM1_ESM.docx]

**Supplementary Information**

**Computational Carrier Dynamics across Heterojunction Interface between Hole Injection and Transport Layers in Quantum-Dot Light-Emitting Diodes**

Sung-Min Jung^a,†^*, Yoonwoo Kim^a^*, Jeong-Wan Jo^a,‡^, Dean M Thelen^b^, Michal Mlejnek^b^, Caicai Zhang^c^ and Jong Min Kim^a^

^a^*Electrical Engineering Division, Department of Engineering, University of Cambridge, 9 JJ Thomson Ave, Cambridge, CB3 0FA, United Kingdom*

^b^*Corning Research and Development Corporation, Corning, New York, 14831, United States of America*

^c^*Corning Incorporated, Corning, New York, 14831, United States of America*

^†^Current address: *AllFocal Optics, St John’s Innovation Centre, Cambridge, CB4 0WS, United Kingdom*

^‡^Current address: *Department of Electronics Engineering, Incheon National University, Incheon 22012, Republic of Korea*

**Quantitative metrics for simulation–experiment comparison**

The RMSE of the current density and the EQE are defined by the following equations.

,	(S1)

$$\mathrm{RMSE}_{J}=\sqrt{\frac{1}{N}\sum_{i=1}^{N} \left( J_{i}^{\exp}-J_{i}^{\mathrm{sim}} \right)^{2}}$$

,	(S2)

$$\mathrm{RMSE}_{\mathrm{EQE}}=\sqrt{\frac{1}{N}\sum_{i=1}^{N} \left( \mathrm{EQE}_{i}^{\exp}-\mathrm{EQE}_{i}^{\mathrm{sim}} \right)^{2}}$$

.	(S3)

$$\mathrm{RMSE}_{\log L}=\sqrt{\frac{1}{N}\sum_{i=1}^{N} \left( {\log_{10}L'}_{i}^{\exp}-{\log_{10}L'}_{i}^{\mathrm{sim}} \right)^{2}}$$

Here, *N* denotes the number of the data points, and *N* = 21 in this analysis, and $L'$ represents the dimensionless luminance normalised by 1 cd m^-2^, i.e., $L^{'}= L/(1 cd m^{-2})$, which preserves the physical scale while allowing a logarithmic comparison. Using the voltage-aligned approach, the RMSE values were calculated as 34.70 mA cm⁻² for current density, 0.388 for luminance, and 0.479% for EQE. The mean absolute percentage deviation is defined by Eq. (R6) for each physical quantity.

.	(S4)

$$PD[\%]=\frac{100}{N}\sum_{i=1}^{N} \left| \frac{y_{i}^{\exp}-y_{i}^{\mathrm{sim}}}{y_{i}^{\exp}} \right|$$

Figure S1. Stability test of the electron density based on drift-current density models at the HIL/HTL interface. Time-dependent electron densities at (a) the 20^th^ nodal point on the HIL boundary, *n*_20_, and (b) the 21^st^ nodal point in the HTL domain, *n*_21_, for the mean value scheme. Time-dependent hole densities of (c) *n*_20_ and (d) *n*_21_ for the field-dependent carrier density selection scheme.

Figure S2. Emission spectrum of the CdSe/ZnS red QD-LED device under the exciton source of 400 nm peak wavelength.

Figure S3. Simulated charge transport characteristics of QD-LED devices using a field-dependent carrier density selection scheme under applied voltages from 1 V to 6 V in 1V increments. (a) Potential distributions, (b) charge density distributions, (c) drift-diffusion current density distributions, and (d) hole and electron current densities across the device.

Figure S4. Comparison between experiments and simulations for (a) current densities, (b) luminance, and (c) EQE curves according to the applied voltage.

Figure S5. Simulated charge transport characteristics of QD-LED devices using a field-dependent carrier density selection scheme for different grid spacing of Δ*z*_HTL_ = 0.25 nm, 0.5 nm, 1.0 nm, and 2.0 nm. (a) Energy level distributions, (b) potential distributions, (c) electric-field distributions, and (d) hole and electron current densities, across the device.

Figure S6. Computational time required for different grid spacing (Δ*z*_HTL_ = 0.25 nm, 0.5 nm, 1.0 nm, and 2.0 nm). The total computation time was evaluated for charge transport simulations conducted under forward bias voltages ranging from 1.0 V to 6.0 V in 0.1 V increments, with each voltage step integrated up to 10 μs to ensure a steady-state solution. Simulations were performed on a Windows platform equipped with an Intel® Core™ i9-7940X CPU operating at 3.10 GHz.

Figure S7. Charge transport simulation results for QD-LED devices with different valence band energy offsets Δ*E*_V_^(HIL-HTL)^ at the HIL/HTL interface: 0.00 eV, 0.15 eV, 0.30 eV, 0.45 eV, and 0.60 eV. (a) Potential distribution and (b) electric-field intensity distribution around the HIL/HTL interfaces. (c) Charge density distributions across the device. (d) Electric-field distributions in the EML.

Figure S8. Simulated charge transport behaviours for QD-LED devices with different doping densities in the HIL layer *N*_A_^HIL^: 2.0×10^19^ cm^-3^, 4.0×10^19^ cm^-3^, 6.0×10^19^ cm^-3^, 8.0×10^19^ cm^-3^, and 1.0×10^20^ cm^-3^. Potential distributions in (a) full scale and (b) magnified scale. (c) Charge density distribution.

**Table S1. Materials and device parameters of transport layers and electrodes used in the simulation.**

| **Parameters** | **Anode**  (ITO) | **HIL**  (PEDOT:PSS) | **HTL**  (TFB) | **ETL**  (ZnO) | **Cathode**  (Al) |
| --- | --- | --- | --- | --- | --- |
| Thickness [nm] |  | 20 | 40 | 40 | - |
| Dielectric constant *ε*_r_ |  | 3.0 ^[1]^ | 9.4^a^ | 9.4^a^ | - |
| Hole mobility *μ*_p_ [cm^2^ V^-1^ s^-1^] |  | 0322×10^–3 [1]^ | 2.0×10^–3 [2]^ | - | - |
| Electron mobility *μ*_n_ [cm^2^ V^-1^ s^-1^] |  | - | - | 2.0×10^–3 [3]^ | - |
| Doping type |  | *p*-type | *p*-type | *n*-type |  |
| Doping density *N*_A_ or *N*_D_ [cm^-3^] |  | 2.81×10^19 [4]^ | 1.00×10^17 [5]^ | 1.00×10^17 [5]^ | - |
| Intrinsic carrier density *n*_i_ [cm^-3^] |  | 1.63×10^6^ | 1.59×10^-6^ | 6.94×10^-10^ |  |
| Work functions *ϕ* [eV] | 4.70 ^[6]^ | - | - | - | 4.00 ^[7]^ |
| Conduction band edge *E*_C0_ [eV] |  | -3.58 ^[8]^ | -2.60 ^[9]^ | -4.00 ^[7,10]^ | - |
| Valance band edge *E*_V0_ [eV] |  | -5.15 ^[8]^ | -5.60 ^[11]^ | -7.40 ^[7.10]^ | - |

^a^Dielectric constants of TFB and ZnO are equally set with that of QDs to avoid the field-mismatch for the charge balance between hole and electron injection at HTL/QD and QD/ETL interfaces.

**Table S2. List of material parameters for QDs used in the simulation.**

| **QD Parameters** | **Core (Shell)** |
| --- | --- |
| Diameter / Shell thickness [nm] | 9.0 (0.5) |
| Number of QD layers | 2 |
| Dielectric constant *ε*_r_ | 9.4 ^[12]^ |
| Hole mobility *μ*_p0_^QD^ [cm^2^ V^-1^ s^-1^] @ *F*_0_ = 5.0×10^6^ [V cm^-1^] | 2×10^-6 [13]^ |
| Electron mobility *μ*_n0_^QD^ [cm^2^ V^-1^ s^-1^] @ *F*_0_ = 5.0×10^6^ [V cm^-1^] | 2×10^-6 [13]^ |
| Peak wavelength *λ*_0_ [nm] | 630 |
| Full width at half maximum [nm] | 20 |
| Conduction band edge *E*_C0_ [eV] | - 3.82^a^ (-3.35) |
| Optical bandgap *E*_G_ [eV] | 1.97^a^ (2.90 ^[14]^) |
| Langevin radiative recombination strength *B* [cm^3^ s^-1^]^c^ | 0.77×10^-12 [15]^ |
| SRH non-radiative recombination lifetime *τ*_p_ = *τ*_n_ [µs]^c^ | 129 |
| Auger non-radiative recombination probability *C*_p_ = *C*_n_ [cm^6^ s^-1^]^c^ | 3.1×10^-31^ |
| Optical efficiency of QD-LED device *η*_opt_ | 9.8% |
| Charge injection coefficient *S*_p_ = *S*_n_ for the hole and electron | 0.46 |

^a^The conduction band edge of QD core and shell is obtained by Eq. (S5) for the charge balance condition.

(S5)

$$E_{C0}=\frac{1}{2}\left( E_{V0}^{H}+E_{C0}^{E} \right)+\frac{1}{2}E_{G}$$

The optical bandgap of QDs *E*_G_ is calculated from *E*_G_ = *hc*/*λ*_0_ for the Planck constant *h*, the speed of light *c* and peak wavelength *λ*_0_ of light emitted from QD.

**Table S3. Direct numerical stability test.**

| Δ*z*_HTL_ [nm] | Δ*t* [ps] | *C* | *J* [A cm^-2^] | Convergence |
| --- | --- | --- | --- | --- |
| 0.25 | 0.5 | 0.041 | 0.544 | O |
| 0.25 | 1.0 | 0.083 | 0.544 | O |
| 0.25 | 5.0 | 0.414 | - | X |
| 0.25 | 10 | 0.828 | - | X |
| 0.50 | 0.5 | 0.010 | 0.544 | O |
| 0.50 | 1.0 | 0.021 | 0.544 | O |
| 0.50 | 5.0 | 0.103 | - | X |
| 0.50 | 10 | 0.207 | - | X |
| 1.00 | 0.5 | 0.003 | 0.540 | O |
| 1.00 | 1.0 | 0.005 | 0.540 | O |
| 1.00 | 5.0 | 0.026 | 0.540 | O |
| 1.00 | 10 | 0.052 | 0.540 | O |
| 2.00 | 0.5 | 0.001 | 0.527 | O |
| 2.00 | 1.0 | 0.001 | 0.527 | O |
| 2.00 | 5.0 | 0.006 | 0.527 | O |
| 2.00 | 10 | 0.013 | 0.527 | O |

**References**

1.	Xu, B. *et al.* Functional solid additive modified PEDOT:PSS as an anode buffer layer for enhanced photovoltaic performance and stability in polymer solar cells, *Sci Rep* **7**, 1-12 (2017).

2.	Zhao, Y. *et al.* Composite hole transport layer consisting of high-mobility polymer and small molecule with deep-lying HOMO level for efficient quantum dot light-emitting diodes, *IEEE Electron Device Lett* **41**, 80–83 (2020).

3.	Wang, F. *et al.* Achieving balanced charge injection of blue quantum dot light-emitting diodes through transport layer doping strategies, *J Phys Chem Lett* **10**, 960–965 (2019).

4.	Yan, F., Parrott, E. P. J., Ung, B. S.-Y. & Pickwell-MacPherson, E. Solvent doping of PEDOT/PSS: Effect on terahertz optoelectronic properties and utilization in terahertz devices, *J Phys Chem C* **119**, 6813–6818 (2015).

5.	Kumar, B., Campbell, S. A. & Ruden, P. P. Modeling charge transport in quantum dot light emitting devices with NiO and ZnO transport layers and Si quantum dots, *J Appl Phys* **114**, 1–6 (2013).

6.	Park, Y., Choong, V., Gao, Y., Hsieh, B. R. & Tang, C. W. Work function of indium tin oxide transparent conductor measured by photoelectron spectroscopy, *Appl Phys Lett* **68**, 2699–2701 (1996).

7.	Kim, J. *et al.* ZnO nanowire-embedded Schottky diode for effective UV detection by the barrier reduction effect, *Nanotechnology* **21**, 115205 (2010).

8.	Vahabzad, F., Rostami, A., Dolatyari, M., Rostami, G. & Amiri, I. S. Solution-processed QD-LEDs in visible range: Modulation bandwidth enhancement, *Physica B: Condensed Matter* **574**, 411667 (2019).

9.	Kim, H. et. al. Interlayer doping with p-type dopant for charge balance in indium phosphide (InP)-based quantum dot light-emitting diodes, *Opt Express* **27**, A1287–1296 (2019).

10.	Vallisree, S., Thangavel, R. & Lenka, T. R. Modelling, simulation, optimization of Si/ZnO and Si/ZnMgO heterojunction solar cells, *Mater Res Express* **6**, 025910 (2018).

11.	Kim, T. *et al.* Efficient and stable blue quantum dot light-emitting diode, *Nature* **586**, 385–389 (2020).

12.	Canali, C., Nava, F. & Ottaviani, G. Hole and electron drift velocity in CdSe at room temperature, *Solid State Commun* **11**, 105–107 (1972).

13.	Kathirgamanathan, P., Kumaraverl, M., Bramananthan, N. & Ravichandran, S. High efficiency and highly saturated red emitting inverted quantum dot devices (QLEDs): optimisation of their efficiencies with low temperature annealed sol–gel derived ZnO as the electron transporter and a novel high mobility hole transporter and thermal annealing of the devices, *J Mater Chem C* **6**, 11622–11644 (2018).

14.	Chaurasiay, R. & Dixit, A. Transition metal dopeed ZnS monolayer: The first principles insights, *The Physics of Semiconductor (IWPSD 2017),* *Springer Proceedings in Physics* **215**, 49–56 (2017).

15.	Liang, C. *et al.* Modeling and simulation of bulk heterojunction polymer solar cells, *Sol Energy Mater Sol Cells* **127**, 67–86 (2014).
